# Cost-utility of aripiprazole once-monthly versus paliperidone palmitate once-monthly injectable for schizophrenia in China

**DOI:** 10.1371/journal.pone.0317393

**Published:** 2025-06-26

**Authors:** Yiping An, Gang Fang, Zhipeng Pi, Yumeng Zhang, Wei Li, Jinxi Ding

**Affiliations:** 1 School of International Pharmaceutical Business, China Pharmaceutical University, Nanjing, China; 2 Pharmaceutical Market Access Policy Research Center, School of International Pharmaceutical Business, China Pharmaceutical University, Nanjing, China; Jawaharlal Institute of Postgraduate Medical Education and Research, INDIA

## Abstract

**Objectives:**

From the perspective of Chinese healthcare system, this study compared the cost-utility of aripiprazole once-monthly (AOM) and paliperidone palmitate once-monthly injectable (PP1M) in the treatment of adult patients with schizophrenia in China.

**Methods:**

A 5-state Markov model was developed to evaluate the cost-utility of 10 years of long-acting injections (LAI) treatment for schizophrenia. The long-term costs and quality-adjusted life years (QALYs) were estimated, with the incremental cost-effectiveness ratio (ICER) as the primary outcome. The annual discount rate was set at 5%. A cost-effectiveness threshold (CET) of 0.51 times China’s 2023 gross domestic product (GDP) (US$ 6,394.536) was used to judge the economics of intervention.

**Results:**

The current price of AOM in China is relatively high (US$418.140). To assess its cost-effectiveness in the context of potential price negotiations with China Healthcare Security Administration (CHS) for inclusion in the National Reimbursement Drug List (NRDL), we simulated a 40% price reduction (US$257.619). At a CET of 0.51 times GDP per capita (US$6,394.536), the base-case analysis showed that the incremental costs of AOM relative to PP1M after 10 years of treatment were US$1,926.373 with an incremental gain of 0.306 QALYs. The ICER for AOM was US$6,285.303 per QALY, which is below the CET, indicating that AOM is cost-effective. One-way sensitivity analysis identified AOM’s drug cost as the parameter with the greatest impact on results. Probabilistic sensitivity analysis revealed that with a 40% price reduction, the probability of AOM being cost-effective is only 41.70%. However, with a 60% price reduction, AOM became dominantly cost-effective, with the probability increasing to 100%. When the CET was relaxed to 0.90 times GDP per capita (US$11,284.476), the probability of cost-effectiveness for AOM after a 40% price reduction rose to 85.10%. Scenario analyses conducted over a time horizon extending from 10 to 30 years showed that the ICER decreased significantly with longer follow-up, gradually approaching the 0.51GDP threshold and remaining below the 0.90 GDP threshold throughout the analysis.

**Conclusions:**

The cost-effectiveness of AOM relative to PP1M is highly influenced by its price and the CET. Healthcare decision makers or clinical users need to balance innovation incentives and accessibility.

## Introduction

The World Health Organization (WHO) reports that schizophrenia affects approximately 24 million people globally [1]. Meanwhile, ‘The National Information System for Psychosis’ indicates that the condition impacts about 4.6 million individuals in China [2]. An epidemiological study covering 195 countries and territories found that China has one of the highest prevalence rates in the world [3]. Schizophrenia often develops in young adults in their twenties, severely affecting personal functions and exacerbating the loss of productivity in an ageing society.

Schizophrenia has a high disease burden. It is characterized by poor treatment adherence and relapse is common. Key factors affecting adherence and relapse include the illness duration, number of relapses, patients’ treatment beliefs, and the presence of family members or caregivers. In outpatient or post-discharge settings, patients often lack continuous supervision, and some families may conceal the diagnosis. Longitudinal data show that the longer the follow-up period, the poorer the patient compliance and the higher the relapse rate, the three-year compliance rate was only 40.87%, and the three-year relapse rate was reported as 61.74%.[4] A disease history of multiple relapse is associated with a poorer prognosis for patients, indicating that patients require a longer treatment period to achieve the same efficacy as before the relapses [5], which exacerbates the burden on patients. Therefore, sustaining long-term treatment to prevent relapse is critical in the management of schizophrenia.

The latest Chinese clinical guideline recommends that atypical antipsychotic LAI should be applied as first-line strategy for both the acute and maintenance treatment schizophrenia. Patients who initiate LAI treatment early in the course of illness experience significantly lower rate of hospitalization and treatment discontinuation compared to those treated with oral antipsychotics [6].

AOM is the newest third-generation atypical LAI launched in China in 2023, and the world’s first dopamine D2 and 5HT-1A receptors partial agonist. In contrast, second-generation LAI such as paliperidone and risperidone are full antagonists of 5HT-2A and D2 receptors. In terms of mechanism of action, partial agonists bind more strongly to the D2 receptors than full antagonists, which makes them more effective at ameliorating positive symptoms and the risk of high prolactin levels. Moreover, partial agonists also improves negative, cognitive, and affective symptoms, whereas full antagonists are primarily ameliorate positive symptoms [7]. AOM achieves a balance of agonistic and antagonistic effects, making it a dopamine system stabilizer. A positively controlled (AOM vs oral aripiprazole), randomized, double-blind phase III marketed clinical trial [8] (NCT03172871) demonstrated the efficacy and safety of AOM in Chinese acute schizophrenia patients.

PP1M was launched in China in 2011 and has been included in the China NRDL in 2020. A post-marketing phase IV single-arm, open-label clinical trial was conducted in China to evaluate its use in patients with acute schizophrenia [9]. PP1M is highly similar to AOM. They have the same indication, administration route, dosing frequency and treatment sequence. Furthermore, PP1M is considered a standard therapeutic drug, and is recommended by both Chinese and international guidelines [10–12]. Therefore, this study aims to evaluate the cost-utility of AOM compared with PP1M in treating Chinese adult schizophrenia from the perspective of the Chinese healthcare system.

## Methods

### Study design

**Fig 1:**
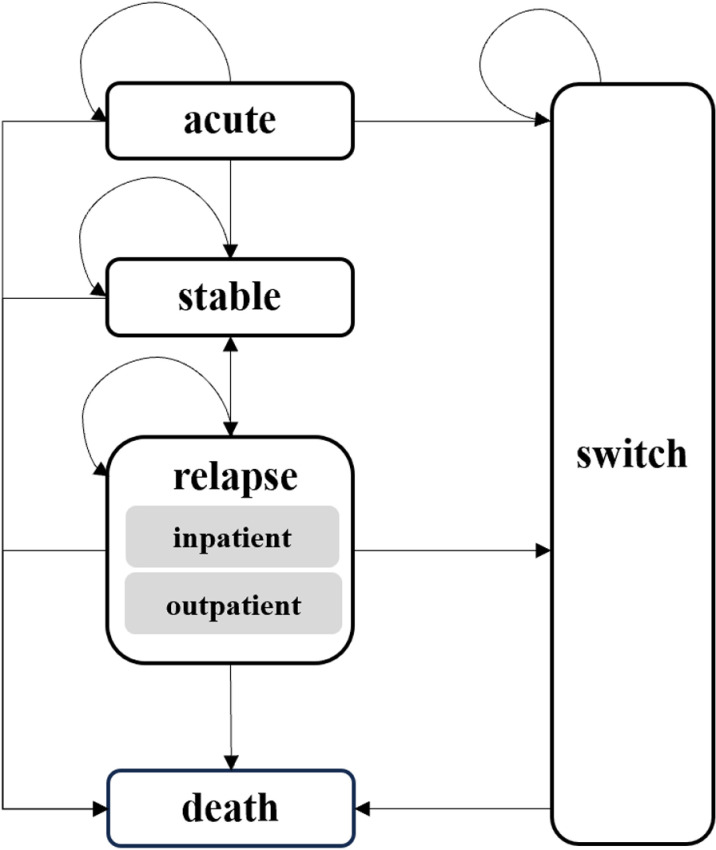
Markov model. The model includes five health states, represented by black rectangular boxes. The relapse state is subdivided into outpatient and inpatient populations. Arrows indicate possible transitions between health states, which occur in monthly cycles.

Compared with the decision tree model, the Markov model demonstrates the disease course in schizophrenia more completely, which is consistent with the characteristics of chronic diseases and long-acting injectable drugs. We used Excel 2021 to build a 5 state-transition Markov model (Fig 1) to evaluate the cost-utility of AOM and PP1M over a 10-year horizon. A matching‐adjusted indirect comparisons (MAIC) was performed between the AOM Phase III Trial and the PP1M Phase IV Trial to derive acute efficacy and safety data. A meta-analysis of other placebo-controlled randomized clinical trials of AOM and PP1M was conducted to obtain relapse rate. Additional transition probability parameters were obtained from published literature. Only the direct medical costs were considered, all costs are based on the official exchange rate of US$1 = 7.127 yuan. (The People’s Bank of China on June 30, 2024) [13] The mapping method and published literature were used to obtain utility values. The outcome of this study was ICER, which represents the costs of a schizophrenia patient per additional healthy year of survival. According to the current pharmacoeconomic evaluation guidelines in China, the annual discount rate for costs and utilities was set at 5% [14]. According to a cost-effectiveness threshold study covering 174 countries, the recommended threshold for China is 0.51 (0.37–0.90) times GDP [15]. Therefore, our study sets the cost-effectiveness threshold at $6,394.536 (0.51 times the per capita GDP of China in 2023) [16]. The results of the probabilistic sensitivity analysis are also presented for thresholds of 0.37 and 0.90 times GDP.

### Patient population

Patient characteristics were matched to the baseline patient cohort from our MAIC result. (See appendix 3 in the S1 File for details of MAIC.) Patients enter the model at age 31.5 years, with 55% of the cohort being male, BMI:23.2 kg/m^2^, Weight: 64.4 kg, Han Ethnicity:99%, PANSS total score:91.8, CGI-S total score:5.3, PSP total score:44.9.

### Model structure

The Markov model consists of five independent health states: (1) Acute, which is the initial state for all patients, is set to 12 weeks according to the timeframe of the AOM phase III trial study, and all patients are transferred out of this state after 12 weeks; (2) Stable; (3) Relapse, which includes both outpatient and inpatient; (4) Switch; (5) Death. The classification of health states is based on the ‘Diagnostic and Treatment Guidelines for Mental Disorders (2020 Edition)’, [17] which categorizes schizophrenia into four treatment states: acute, consolidation, maintenance, and chronic. (See appendix 1 in the S1 File for details of the definitions of the health states and treatment modalities.)

The doses of the therapeutic drugs were derived from the product instructions. The choice of drugs for the switch state was informed by the consensus of clinical experts interviewed. (See appendix 5 in the S1 File for details of clinical research.) They considered patients who transition from relapse to switch state to be more severely ill than those who transition from acute to switch state, and therefore these two states of patients are treated with different oral antipsychotics, with olanzapine being the last treatment option. A 1-month cycle was used based on the injection times specified in the instructions for AOM and PP1M. Half-cycle corrections were applied to each cycle to adjust the costs and QALYs to occur at the midpoint of the month.

### Clinical inputs and transition probabilities

This study incorporated clinical parameters and transition probabilities derived from a combination of direct trial evidence, indirect comparisons, meta-analyses, and published literature to reflect real-world clinical pathways of schizophrenia treatment. Given the lack of comprehensive head-to-head data, a MAIC and additional meta-analytical approaches were applied to estimate responder, relapse, mortality, and adverse events rates. (See appendix 2 in the S1 File for summarized model inputs.)

The QUALIFY study is the only head-to-head clinical trial comparing AOM and PP1M that focused on quality of life [18]. However, its primary outcomes, the Heinrichs-Carpenter Quality of Life Scale (QLS) scores and the Clinical Global Impression-Severity scale (CGI-S), are not directly applicable for estimating transition probabilities. Although these disease-specific scale scores are useful for precise clinical assessment, they are difficult to interpret in terms of clinical significance, for example, it is unclear what a one-point improvement on a given scale means for a patient’s real-world functioning. In this study, we converted the continuous variables from such scales into binary outcomes to derive transition probabilities for the Markov model.

The transition probability for the acute state was based on the responder rate, defined as ≥30% reduction in the Positive and Negative Syndrome Scale (PANSS) total score [8]. This parameter was derived from the MAIC by adjusting individual patient data (IPD) from the AOM phase III trial and aggregated data (AgD) from the PP1M phase IV Trial. Both trials included patients experiencing acute exacerbations of schizophrenia (baseline PANSS total score ≥70). The unadjusted responder rates were (AOM: 88.600%, PP1M: 82.110%), and the adjusted indirectly comparable responder rates were (AOM: 90.500%, PP1M: 82.110%). (See appendix 3 in the S1 File for details of MAIC.)

Due to the limited duration of the AOM phase III trial (12 weeks) and the PP1M phase IV trial (13 weeks), and the absence of efficacy and safety data in the relapse state, this study hypothesized that responder and adverse event rate in the relapse state would be equivalent to those in the acute state. This assumption was endorsed by clinical experts and corroborated by published literature. Emsley et al [19] conducted a 7-year cohort study comparing treatment responder rate, remission rate, and total PANSS scores between patients using LAI for schizophrenia after a first episode and those after a relapse. The results showed no significant differences, leading to the conclusion that the responder rate to treatment in relapses were similar to those in the first episodes.

Since there are no published meta-analyses specifically addressing LAI, for schizophrenia that include relapse rate, we conducted a meta-analysis to estimate the relapse rate in the stable state. (See appendix 4 in the S1 File for details of meta.) This analysis yielded a relative risk (RR) of relapse for AOM versus PP1M of 0.691 (95% CI, 0.407–1.173), with relapse rate (AOM: 3.451%, PP1M: 4.994%). Transition probabilities for relapse in inpatient and outpatient settings were calculated by multiplying the relapse rate by the hospitalization rate(AOM:64.000%, PP1M 51.440%) [20].

The mortality rate for the general population was derived from the latest China Statistical Yearbook and is 0.062% [21]. The transition probability of death state was calculated from the RR values of mortality in schizophrenia patients in the relapse state vs the general population(AOM:3.516, PP1M:3.092), and the RR values of mortality in schizophrenia patients in the stable phase vs the general population.(AOM:2.859, PP1M:2.622) [22].

In addition to the transition probability, the clinical inputs include the rates of adverse events as shown in appendix2. To simplify the model calculations, only three adverse events of particular interest from the AOM phase III trial were considered, including weight gain (defined as weight increased by≥7%), prolactin-related adverse events (included decreased or increased blood prolactin levels, hyperprolactinemia), and extrapyramidal symptoms (EPS)-related adverse events (included dystonia, Parkinson disease, akathisia, muscle rigidity, etc). The adverse event rates for the acute and relapse states were derived from the MAIC. The adverse event rates for the stable and switch states were derived from the QUALIFY study [18]. This reported the aforementioned three adverse events in the AOM versus PP1M trial with a follow-up period of up to 32 weeks, which compensated for the short time frame in observing adverse event rates in the AOM phase III trial and the PP1M single-arm trial.

### Costs inputs

Direct medical costs encompass three primary components: drug acquisition costs, drug and disease management costs, and adverse event management costs. A summary of these costs is presented in appendix2.

To support AOM healthcare access decisions, this study provides a reference for price determination. According to our statistics, the price reductions for newly negotiated NRDL drugs in China from 2017 to 2022 ranged approximately from 44.0% to 60.1%. We adopted a conservative estimate, assuming AOM’s price would decrease by at least 40% from the current price (US$418.140/400 mg), resulting in a simulated price of US$257.619/400 mg, for the cost-effectiveness comparison with PP1M.

The latest drug prices for each state treatment modality were obtained from China Pharmaceutical Information Database [23]. Drug and disease management costs were derived from publicly available data and expert research. The frequency of medical resource utilization by patients in different health states was determined based on psychiatrists’ opinions, including psychiatric clinical assessments, the number of psychiatric scale evaluations, hospitalization days, blood and urine tests, and electroencephalograms. (See appendix 6 in the S1 File for details of the costs.) The unit costs of these medical resources were derived from the average unit prices of medical projects published by the governments of five provinces in China.

Adverse events were managed in accordance with the literature [24]. Weight gain was addressed with metformin hydrochloride tablets, prolactin-related adverse events with bromocriptine mesylate tablets, and EPS-related adverse events with phenazopyridine hydrochloride tablets. The treatment duration for each was set at 14 days per cycle, based on expert research.

### Utility inputs

Utility and disutility inputs were shown in appendix2. Currently, there is a lack of schizophrenia utility studies specific to the Chinese population. To utilize the acute state population data from the AOM phase III trial, we employed the mapping method to estimate the acute state utilities. This method involves converting non-utility scale scores to utility scale scores using regression equations [25]. The mapping formula (Formula 1) was derived from a study based on an Asian population, with 59.8% being Chinese, which mapped the PANSS score onto the five-level EuroQol five-dimensional (EQ-5D-5L) utilities [26].


$$\mathrm{\backslash newsavebox}\mathrm{\backslash eqbox}\mathrm{\backslash newlength}\mathrm{\backslash width}\mathrm{\backslash newlength}\mathrm{\backslash height}\mathrm{\backslash newlength}\mathrm{\backslash depth}\begin{array}{cc} EQ-5D-5L\;utility & =1.3103-0.0044\times positive+0.0025\times negative-0.0146\times general\;psychopathology\\ & -0.0029\times age+0.0149\times female \end{array}\mathrm{\backslash settowidth}\mathrm{\backslash width}\mathrm{\backslash usebox}\mathrm{\backslash eqbox}\mathrm{\backslash settoheight}\mathrm{\backslash height}\mathrm{\backslash usebox}\mathrm{\backslash eqbox}\mathrm{\backslash settodepth}\mathrm{\backslash depth}\mathrm{\backslash usebox}\mathrm{\backslash eqbox}\mathrm{\backslash addtolength}\mathrm{\backslash depth}0.5pt\mathrm{\backslash newwrite}\mathrm{\backslash file}\mathrm{\backslash immediate}\mathrm{\backslash openout}\mathrm{\backslash file}=\mathrm{\backslash jobname}.bsl\mathrm{\backslash immediate}\mathrm{\backslash write}\mathrm{\backslash file}Depth=\mathrm{\backslash the}\mathrm{\backslash depth}\mathrm{\backslash immediate}\mathrm{\backslash write}\mathrm{\backslash file}Height=\mathrm{\backslash the}\mathrm{\backslash height}\mathrm{\backslash addtolength}\mathrm{\backslash height}\mathrm{\backslash depth}\mathrm{\backslash immediate}\mathrm{\backslash write}\mathrm{\backslash file}TotalHeight=\mathrm{\backslash the}\mathrm{\backslash height}\mathrm{\backslash immediate}\mathrm{\backslash write}\mathrm{\backslash file}Width=\mathrm{\backslash the}\mathrm{\backslash width}\mathrm{\backslash closeout}\mathrm{\backslash file}\mathrm{\backslash usebox}\mathrm{\backslash eqbox}$$

                                                 Formula 1

positive: positive subscales of PANSS, negative: positive subscales of PANSS, general psychopathology: subscales of PANSS

According to expert opinion, the utilities for the switch state are considered to be the average of the utilities for relapse outpatient and relapse inpatient states. This averaging approach was also employed in the absence of specific utilities for an 8-state Markov model study of schizophrenia [27].

### Model validation

We validated and refined the study design using the health economic quality evaluation tool CHEERS [28]. (See appendix 10 in the S1 File for details of CHEERS Checklist) During model construction, a targeted literature review of existing pharmacoeconomic studies of LAI for schizophrenia was conducted to ensure that the design of this study was generally consistent with previous studies. For internal validation, the discount rate for costs and utilities was set at 0% to ensure that the undiscounted and discounted quality-adjusted life years (QALYs), as well as cost results, were consistent. For external validation, online and offline expert consultations were organized, employing semi-structured questionnaires and face-to-face interviews to gather opinions from psychiatric clinicians. This process aimed to ensure that the health state setting, simulation horizon, and treatment modalities for each state closely reflected clinical reality.

### Sensitivity analyses

A one-way sensitivity analysis was conducted to assess the impact of individual parameter changes on the base-case results. This analysis involved setting upper and lower limits on the model parameters, allowing each parameter to vary individually within 95% confidence intervals reported in the literature or by ±10% of the base-case values (when confidence intervals were not available). The annual discount rate was varied between 3% and 8%. Parameters varied included costs, utilities, and the incidence of adverse events, totaling 45 parameters. The results were depicted in the form of a tornado diagram, with parameters ranked in descending order based on the magnitude of their impact on net monetary benefits (NMB).

A probability sensitivity analysis was conducted using 1000 Monte Carlo simulations. The parameter distributions were specified with reference to ‘Cost-Effectiveness Modelling for Health Technology Assessment’ [29]. Costs and disutility parameters were assumed to follow a Gamma distribution, while utilities and the incidence of adverse events parameters were assumed to follow a Beta distribution. The results were presented in the form of cost-effectiveness acceptability curves (CEACs) and incremental cost-effectiveness scatter plots.

### Scenario analyses

A systematic review and meta-analysis of life expectancy in patients with schizophrenia revealed that the life expectancy of Asian patients was 60.2 years (95% CI: 56.6–63.8) [30]. In contrast, the baseline mean age of patients with AOM and PP1M after MAIC was 31.5 years. Consequently, scenario analyses adjusted the simulation horizon of the study to a maximum of 30 years. The results were presented as line graphs.

## Result

### Base-case analysis

Table 1 presents the results of the base-case analysis. Over a 10-year horizon, the incremental costs of AOM compared to PP1M treatment were US$1,926.373, with an incremental QALY of 0.306 and an ICER of US$6,285.303/QALY. This ICER is below the CET of 0.51 times GDP(US$6,394.536). In the acute state, the total costs of AOM are lower than those of PP1M, and both have a QALY of 0.118, making AOM the dominant strategy in short-term 12-week treatments. In other health states, AOM has a larger population in the stable state, and cumulative treatments generate more QALYs than PP1M. (See appendix 8 in the S1 File for details of cost and utility components).

**Table 1:** Base-case analysis.

	AOM	PP1M
Costs outcomes (US$)		
Drug acquisition costs	25,464.029	19,146.105
Drug and disease management costs	11,684.268	15,549.140
Adverse event management costs	6.853	36.218
Total costs(discounted)	36,494.821	34,568.448
Health outcomes		
Treatment increased QALYs	7.552	7.235
AE decreased QALYs	−0.032	−0.027
Total QALYs (discounted)	7.384	7.077
Incremental results		
Incremental costs (US$)	1,926.373	–
Incremental QALYs	0.306	–
ICER (US$/QALYs)	6285.303	–

Although the loss of QALYs due to adverse events is slightly greater for AOM than for PP1M (AOM: −0.032, PP1M: −0.027), this represents a cognitive reversal of the 0.306 incremental QALYs associated with AOM. Nonetheless, the adverse event management costs and the loss of QALYs have a minimal impact on the overall outcome, as confirmed by previous pharmacoeconomic studies [31]. This study did not include adverse event treatment costs in the model. The incremental QALYs in this study were mainly due to the superior performance of AOM in terms of responder rate, and AOM is also cost-effective in the long term.

### Sensitivity analyses

**Fig 2:**
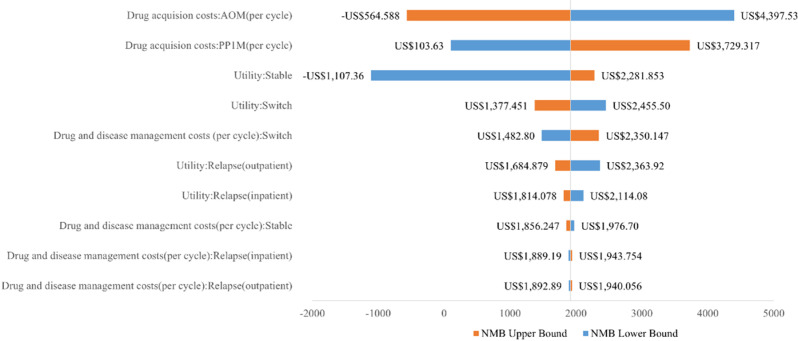
One-way sensitivity analysis. NMB: net monetary benefits. The annual discount rate fluctuates between 3% and 8% and the other parameters fluctuate by ±10%, resulting in a range of variation in the final NMB.

The results of the one-way sensitivity analysis are depicted in Fig 2, which only shows the top 10 parameters influencing the results due to the large number of parameters considered. (See appendix 9 in the S1 File for the results of all 45 parameters.) The parameter exerting the greatest influence on NMB is the cost of AOM drug acquisition, followed by the cost of PP1M drug acquisition. The model used a weighted average price of PP1M based on the market share of the originator and generics, with the originator accounting for over 99% of the market, the overall price of PP1M remains relatively stable, so the model results are primarily influenced by the price of AOM.

**Fig 3:**
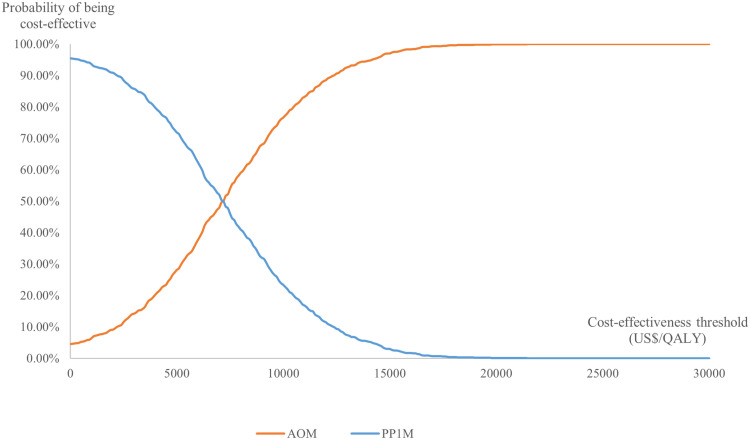
Cost-effectiveness acceptability curve. AOM: aripiprazole once-monthly; PP1M: paliperidone palmitate once-monthly injectable; QALY: quality-adjusted life year.

**Fig 4:**
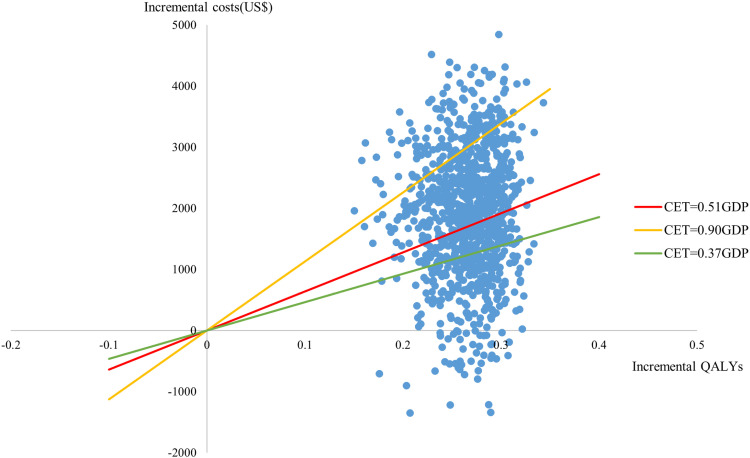
Incremental cost-effectiveness scatter plot. CET: cost-effectiveness threshold; GDP: gross domestic product; 0.37GDP = US$ 4639.173; 0.51GDP = US$ 6394.536; 0.90GDP = US$ 11284.476.

**Fig 5:**
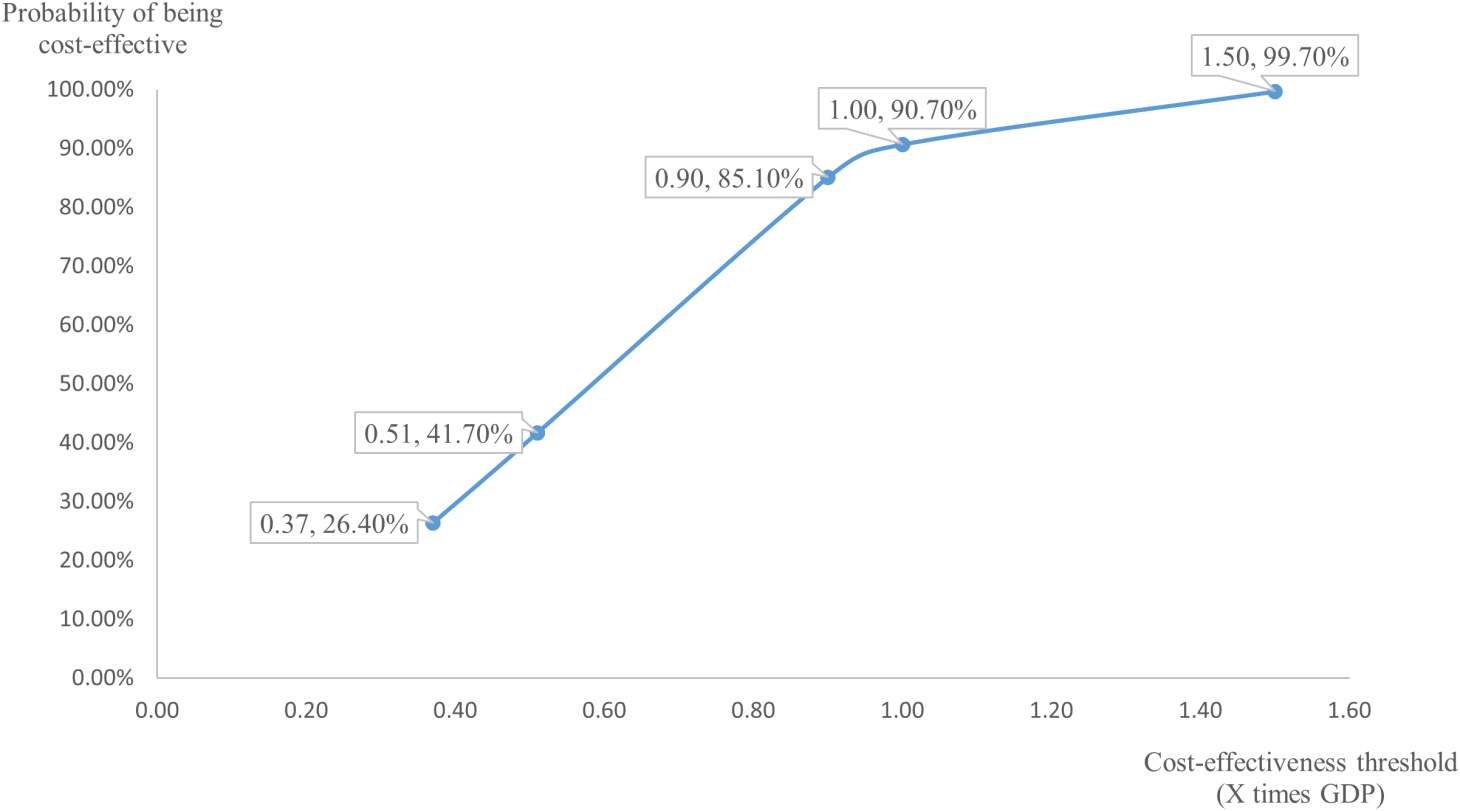
Probability of Cost-Effectiveness Across CET. GDP: gross domestic product.

The results of the probability sensitivity analysis, conducted after 1000 Monte Carlo simulations, are presented in Figs 3 and 4. According to the cost-effectiveness acceptability curve, at the CET of 0.51 times GDP, the probability of AOM being more cost-effective was 41.70%, suggesting that its economic value should be interpreted with caution. As shown in Fig 5, the probability of cost-effectiveness is highly sensitive to changes in the CET within the range of 0.37 to 0.90 times GDP. Beyond a CET of 1.0 times GDP, the rate of increase in probability slows, indicating diminishing marginal gains in cost-effectiveness with further increases in the threshold.

### Scenario analyses

Over a 30-year horizon, the incremental costs of AOM compared to PP1M treatment were US$4,522.573, with an incremental QALY of 0.789 and an ICER of US$5,733.204/QALY. This ICER is below the CET of 0.51 GDP(US$6,394.536). The probability of AOM being cost-effective under this CET was 47.70%. When the CET was relaxed to 0.90 times GDP, the probability increased to 89.70%.

**Fig 6:**
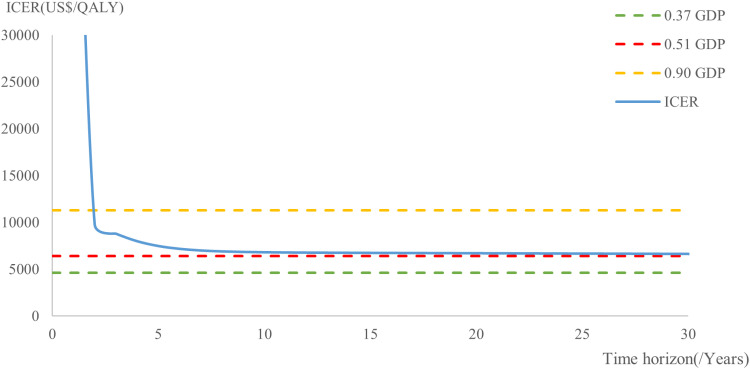
ICER changes with time horizon. GDP: gross domestic product; 0.37GDP = US$ 4639.173; 0.51GDP = US$ 6394.536; 0.90GDP = US$ 11284.476; ICER: incremental cost-effectiveness ratio.

Fig 6 illustrates the trend of ICER under different CET and time horizons. After just one month of treatment, the ICER declined rapidly, then gradually approached the 0.51 GDP threshold and remained below the 0.90 GDP threshold. This trend is driven by the “snowball effect” of ICER accumulation over time and the impact of cost discounting. The use of 0.51 GDP threshold reflects a scenario of severe healthcare resource constraints and may not adequately capture the long-term economic value of AOM when the evaluation period is short. For chronic conditions such as schizophrenia, which often require long-term or lifelong treatment, a moderately higher threshold is recommended.

## Discussion

In this study, we constructed a 5-state Markov model to evaluate the cost-utility of AOM versus PP1M for the treatment of schizophrenia in Chinese adults from the perspective of the Chinese healthcare system. The barrier of lacking head-to-head clinical trial data between AOM and PP1M was broken through a combination of MAIC and meta-analysis. Additionally, the barrier of lacking health utility values for the Chinese population with schizophrenia was broken by employing a mapping approach. These strategies provided Chinese localized parameters for the pharmacoeconomic model and helped fill critical evidence gaps.

We reviewed the current meta-analyses on AOM and PP1M to summarize the existing evidence base on clinical outcomes. There was no meta-analysis focuses exclusively on AOM and PP1M with the formulation restricted to long-acting injectables and schizophrenia currently. Most existing meta-analyses combine various antipsychotic drugs, multiple formulations, and even include patients with psychiatric conditions other than schizophrenia. These analyses generally conclude that long-acting injectables are significantly more effective than placebo, however, they do not demonstrate significant differences in efficacy among different long-acting formulations. For instance, Johannes Schneider-Thoma et al. [32] conducted a large network meta-analysis including 32 antipsychotics, which showed that AOM had a relapse risk ratio (RR) of 0.32 (95% CI: 0.14–0.57) and PP1M had an RR of 0.31 (95% CI: 0.16–0.48) compared to placebo. No statistically significant differences were observed between drugs. Similarly, Giovanni Ostuzzi et al. [33] performed a network meta-analysis of 12 long-acting injectable antipsychotics, reporting that AOM demonstrated better relapse prevention and tolerability than PP1M and ranked highest in tolerability among all 12 drugs. However, the differences were not statistically significant.

Overall, the comparative clinical evidence between AOM and PP1M remains inconclusive, making it difficult for clinicians to distinguish between long-acting injectable options. Our study contributes economic evidence based on existing clinical data to support decision-making in such cases.

We reviewed the pharmacoeconomic evidence for AOM and PP1M to ensure that the conclusions of our study were consistent with those of previous studies. Leslie Citrome et al [1] in the United States constructed a 1-year decision tree model for cost-effectiveness analysis. Clinical inputs were obtained from the placebo-controlled trials of AOM and PP1M [34,35]. The relapse rate in our study was also obtained from these two trials, which are the only available data currently. Their results showed that AOM was dominated at real-world and maximum doses, and the ICER was below the CET at both clinical trial doses and prescription doses. Notably, Leslie Citrome et al. used the relapse rate data from the original trial directly as model inputs, whereas our study conducted a meta-analysis on his basis. Christophe Sapin et al. [36] in the United States constructed a 6-month covariance model for cost-effectiveness analysis. Clinical inputs were obtained from the QUALIFY study. The result demonstrated that AOM was dominant over PP1M in improving QLS and CGI-S scores. A cost-effectiveness analyses in France also based on the QUALIFY study reached the same conclusions [37]. Evanthia Achilla et al [38]. conducted a systematic review of economic evaluation of long-acting/extended-release antipsychotics, including 28 cost-effectiveness studies. They noted that AOM demonstrated economic value primarily through reducing relapse and hospitalization-related costs, while the cost of managing adverse events accounted for only a small proportion of total treatment expenses. These findings are consistent with the cost component results presented in our study.

The economic evidence pertaining to AOM and PP1M is relatively limited. The existing studies were conducted in developed countries and focused on short-term cost-effectiveness analysis with a time horizon of 6 months to 1 year. The primary clinical inputs were derived from the QUALIFY study, which recruited participants from 10 developed countries, and the effect outcomes CGI scores and QLS scores were not conducive to the interpretation of pharmacoeconomic results (costs per subscale score improvement). These evidences are less applicable in developing countries.

In terms of setting the CET, the WHO has recommended a range of 1−3 times GDP per capita, which is widely used in health economic evaluations. However, recent empirical studies have shown that this threshold may be too high and could present potential drawbacks. The threshold was based on the Value of a Statistical Life (VSL) and fails to account for health opportunity costs [39], the loss of health when health expenditures on new treatments crowd out funding for other treatments. Furthermore, excessively high thresholds may reduce overall population health and have limited generalizability across most countries. A study of CET covering 174 countries found that 97% of countries had CET of less than 1 x GDP per QALY, suggesting that China’s threshold could be 0.51 (0.37–0.90) times GDP [15]. In addition to this study, several other relevant empirical studies in China are worth noting. Jessica Ochalek et al [40]. estimated the CET in China based on the marginal productivity of health expenditure from the supply-side threshold, accounting for health opportunity costs. They found that the CET per DALY was 0.63 (0.47–0.88) of GDP per capita in 2017. Dan Cai et al [41]. estimated the CET in China based on the VSL from the demand-side threshold, showing that the CET per QALY was 1.45 (1.36–1.55) times GDP per capita in 2010.

Due to the use of different measurement methods and perspectives, the CET varies considerably. In China’s practice of negotiating access to new drugs for health insurance, different thresholds are applied to drugs in different therapeutic areas such as chronic or rare diseases. The use of 0.51 times GDP as the CET in our study represents a more conservative estimate. Given that China’s total health expenditure in 2023 increased by 6.15% compared to 2022 [42], this threshold could be moderately higher in decision-making.

Our study represents the first long-term cost-utility analysis conducted within the context of a developing country’s healthcare system, with a study horizon of up to 30 years for AOM and PP1M. Clinical inputs for the acute state were derived from the Chinese population for both the AOM and PP1M trials, and acute state utilities were derived from a mapping study that also included a Chinese population. Although the clinical inputs and utilities in other health states were sourced from foreign populations, we have used the available Chinese population data for model inputs as much as possible to make the model more relevant to the Chinese reality. We used utility analysis to make the results more understandable to policy makers (costs per healthy year of life).

However, this study also has the following limitations: first, the use of model parameters was limited by the scarcity of data from head-to-head clinical trials of AOM and PP1M and the short study time frame. In this study, we chose the AOM phase III positive double-blind controlled trial and the PP1M phase IV single-arm open-label trial for MAIC to obtain acute state parameters, and there were differences in the design of the clinical trials themselves, and there was some bias between the results after MAIC and the reality. Most clinical studies have observed fewer relapse events in patients using LAI during the follow-up period, and there is a lack of data for the relapse population. This study hypothesized that the efficacy and safety of the relapse state would be consistent with those of the acute state, this hypothesis requires validation with data from a broader relapse population.

Second, in the meta-analysis of stable state relapse rate, we targeted the specifications of AOM and PP1M as 1-month injections. Only two clinical trials met the requirements after rigorous screening according to the inclusion and exclusion criteria and bias evaluation, which is a weak level of evidence. The current large scale meta-analysis in the field of psychiatric disorders often combined bipolar disorder other than schizophrenia, depression, and other disorders, and included LAI and oral dosage forms and different specifications of drugs. These have limited reference value for the present study.

Third, although we localized the utility value for the acute phase using a mapping approach, the source of the mapping data is based only on the IPD of an AOM phase III trial. This restricts the generalizability of the mapped utility estimate. Moreover, most utility parameters were derived from studies conducted in non-Chinese populations, which limits the external validity of the model results. While we conducted sensitivity analyses for this, variability between populations may still lead to bias. There are fewer acute state utility studies, and the acute state utilities used in published pharmacoeconomic studies were not clearly sourced, so we did not refer to their value for a scenario analysis of different utility sources.

Fourth, both AOM and PP1M are LAI, so the difference in adherence between the two drugs was not considered in this study, however, for the clinical reality of long-term treatment, patients still discontinue their medication. The model was simplified by not accounting for treatment discontinuation rates, which may have led to an overestimation of both overall effectiveness and treatment costs to some extent.

We have tried our best to narrow these gaps through reasonable hypotheses and study design, but it still leads to limited extrapolation of the conclusions of this study. We hope that more long-term prospective trials or retrospective data will be available in the future to demonstrate the clinical differences between the two drugs, and we also hope that there will be local Chinese studies on the health-related quality of life of schizophrenia patients.

Mental health has profound societal and individual impacts. Given the current lack of clear comparative clinical evidence, the choice of long-acting injectable antipsychotics should not rely solely on drug price. Clinical decision-making and reimbursement policies must strike a balance among cost-effectiveness, accessibility, and incentives for pharmaceutical innovation. A tiered pricing strategy, combined with flexible cost-effectiveness thresholds, is recommended as a core approach. In the short term, strict cost control could improve the efficiency of healthcare funding. Over the longer term, thresholds may be moderately relaxed for innovative therapies that address unmet clinical needs or significantly extend survival. Risk-sharing agreements based on therapeutic outcomes should be implemented, and real-world data on AOM and PP1M should inform a re-evaluation mechanism for pharmacoeconomic value two years post-listing, with adjustments to reimbursement prices made accordingly.

Although LAI for schizophrenia have been put into clinical use in China for years, the clinical utilization rate is only 0.66%, much lower than the Asian average of 17.9% [43]. We hope that this study will support the AOM health care access decision, promote the widespread clinical use of LAI, and provide evidence-based guidance for clinical prescribing, improve mental health management in China, and offer a reference for healthcare decision-making in other developing countries.

## Conclusion

The cost-effectiveness of AOM compared to PP1M is highly sensitive to both its own pricing and the selected CET. Under a conservative threshold of 0.51 times GDP, a 40% price reduction results in only modest cost-effectiveness (41.70%), which may fall short of the efficiency standards required for inclusion in the NRDL. However, at a 60% price reduction, AOM achieves 100% cost-effectiveness. When the threshold is moderately relaxed to 0.90 times GDP, a 40% price reduction yields more robust results (85.1%), and the ICER for both 10-year and 30-year treatment horizons fall below the threshold, indicating sustained long-term economic value relative to PP1M.

If policymakers prioritize innovation incentives for AOM, a more lenient threshold may be justified with a smaller price reduction, though it is recommended that the threshold remain below 1x GDP. Conversely, if improving accessibility to long-acting injectables is the main goal, a larger price reduction may be required, with a more constrained threshold.

## Supporting information

S1 File This is the S1 File: Cost-utility of aripiprazole once-monthly versus paliperidone palmitate once-monthly injectable for schizophrenias in China. (DOCX)
