# Supplementary material for: Cost-utility of aripiprazole once-monthly versus paliperidone palmitate once-monthly injectable for schizophrenia in China
Source: PLoS One. 2025 Jun 26;20(6):e0317393. doi: 10.1371/journal.pone.0317393 (PMC12200646; doi:10.1371/journal.pone.0317393)
Supplement: S1 File — (DOCX) [file pone.0317393.s001.docx]

**Cost-utility of aripiprazole once-monthly versus paliperidone palmitate once-monthly injectable for schizophrenia in China**

**Supporting information**

[**Appendix 1 Health state definitions and treatment modalities** 2](#_Toc197936485)

[**Appendix 2 Model inputs** 3](#_Toc197936486)

[**Appendix 3 Matching-Adjustment Indirect Comparison** 6](#_Toc197936487)

[**Appendix 4 Meta-analysis of relapse rate** 8](#_Toc197936488)

[**Appendix 5 Clinical research** 11](#_Toc197936489)

[**Appendix 6 Cost inputs** 12](#_Toc197936490)

[**Appendix 7 Mapping in acute state utility** 17](#_Toc197936491)

[**Appendix 8 Cost and utility components** 17](#_Toc197936492)

[**Appendix 9 One-way sensitivity analysis** 18](#_Toc197936493)

[**Appendix 10 CHEERS 2022 Checklist** 20](#_Toc197936494)

[**References** 23](#_Toc197936495)

**Appendix 1 Health state definitions and treatment modalities**

Refer to AOM and PP1M related clinical trials for definitions of each health state.

Supplementary Table 1 Health State Definition

| **State** | **Definition** |
| --- | --- |
| Acute | Schizophrenia patients with an acute episode, the following conditions need to be met simultaneously：  ①Positive and Negative Syndrome Scale (PANSS) total score ≥ 70；  ②Specific psychotic symptoms on the PANSS with a score ≥ 4 for at least in 2/4 specific items: conceptual, disorganization(P2), hallucinatory, behavior(P3), suspiciousness/persecution(P6), unusual thought content(G9).[1] |
| Stable | Schizophrenia patients with stable condition, the following conditions need to be met simultaneously：  ①Outpatient status;  ②PANSS total score ≤80;  ③Lack of specific psychotic symptoms on the PANSS, as measured by a score of ＜4 (moderate) on each of the following items: conceptual disorganization (P2), suspiciousness (P6), hallucinatory behavior (P3), and unusual thought content (G9);  ④Clinical Global Impressions-Severity (CGI-S)score of ＜4 (moderately ill);；  ⑤Clinical Global Impressions-Severity of Suicidality (CGI-SS) score of ＜2 (mildly suicidal) on part 1 and ＜5 (minimally worsened) on part 2.[2] |
| Relapse | Disease recurrence, long-acting injection-treated patients, divided into outpatient and inpatient conditions, meet either:  ①Clinical Global Impression-Improvement (CGI-I)score of ≥5 (minimally worse)；  ②CGI-SS score of 4(severely suicidal) or 5(attempted suicide)on part 1 and/ or 6 (much worse) or 7 (very much worse) on part 2；  ③Violent behavior resulting in clinically significant self-injury, injury to another person, or property damage[2] |
| Switch | Patients are referred to the next line of treatment for a variety of reasons, including poor effectiveness and intolerance. |

Supplementary Table 2 Treatment of each state

| **State** | **Treatment** |
| --- | --- |
| Acute | AOM group: Aripiprazole tablets (10mg/day,17days)+AOM（400mg/month）  PP1M group: Paliperidone extended-release tablets (3mg/day, 3day) + PP1M (day1: 150mg, day8: 100mg) |
| Stable | AOM group: AOM (400mg/month)  PP1M group: PP1M (75mg/month) |
| Relapse | AOM group: AOM (400mg/month)  PP1M group: PP1M (75mg/month) |
| Switch | Acute to switch: oral antipsychotic (AOM and PP1M group)^a^  Relapse to switch: oral clozapine (AOM and PP1M group) |
| Death | / |

a: Oral antipsychotics include the following 10, according to the market share ranking on menet.com: ①olanzapine tablets, ②paliperidone extended-release tablets, ③quetiapine fumarate tablets, ④aripiprazole tablets, ⑤olanzapine orally disintegrating tablets, ⑥quetiapine fumarate extended-release tablets, ⑦risperidone tablets, ⑧piperolimus hydrochloride tablets, ⑨buprenorphine tablets, ⑩risperidone oral solution.

**Appendix 2 Model inputs**

**2.1 Transition probability inputs**

All parameters were transformed and input into the model using Formula 1. [3]

rate$=-\frac{\ln\left( 1-p1 \right)}{t1}$，p2$=1-e^{-rt2}$

**Formula 1**

rate: rate of occurrence of the event

p1: probability of an event occurring within the time frame of the study as reported in the original literature

t1: time frame for events reported in the original literature

p2: transition probability

t2: cycle period

Supplementary Table 3 Transition probability

| **State** | **Parameters** | **AOM** | **PP1M** | **References** |
| --- | --- | --- | --- | --- |
| Acute to stable | responder rate  in acute state | 90.500% | 82.110% | MAIC |
| Relapse to stable | responder rate  in relapse state | 54.371% | 43.653% | MAIC |
| Acute to switch | switch rate  in acute | 9.500% | 17.890% | 1- responder rate  in acute state |
| Relapse to switch | switch rate  in relapse | 0.140% | 0.140% | [4] |
| Stable to relapse | relapse rate | 3.451% | 4.994% | Meta |
| Stable to relapse | hospitalization rate | 64.000% | 51.440% | [5] |
| Stable to relapse | relapse rate of inpatient | 2.209% | 2.569% | relapse rate$\times$  hospitalization rate |
| Stable to relapse | relapse rate of outpatient | 1.242% | 2.425% | relapse rate$\times$  (1-hospitalization rate) |
| All to death | mortality rate | 0.062% | 0.062% | [6] |
| Stable to death | RR of stable patients vs. general population | 2.859 | 2.859 | [7] |
| Relapse to death | RR of relapse patient vs. general population | 3.516 | 3.516 | [7] |

**2.2 Clinical & Cost & Utility inputs matrix**

Supplementary Table 4 Model inputs

| **Parameters** | **Base-case value** | **Range** | **Distribution** | **References** |
| --- | --- | --- | --- | --- |
| **Clinical inputs** |  |  |  |  |
| AE rates in acute |  |  |  |  |
| Weight gain-AOM | 23.200% | 20.880%~25.520% | Beta | MAIC |
| Weight gain-PP1M | 7.222% | 6.500%-7.944% | Beta | MAIC |
| Prolactin-related -AOM | 1.300% | 1.170%-1.430% | Beta | MAIC |
| Prolactin-related -PP1M | 1.385% | 1.247%-1.524% | Beta | MAIC |
| EPS-related -AOM | 40.600% | 36.540%-44.660% | Beta | MAIC |
| EPS-related -PP1M | 11.037% | 9.933%-12.141% | Beta | MAIC |
| AE rates in relapse |  |  |  |  |
| Weight gain-AOM | 8.423% | 7.581%-9.265% | Beta | MAIC |
| Weight gain-PP1M | 2.468% | 2.221%-2.715% | Beta | MAIC |
| Prolactin-related -AOM | 0.435% | 0.392%-0.479% | Beta | MAIC |
| Prolactin-related -PP1M | 0.464% | 0.418%-0.510% | Beta | MAIC |
| EPS-related -AOM | 15.939% | 14.345%-17.533% | Beta | MAIC |
| EPS-related -PP1M | 3.823% | 3.441%-4.206% | Beta | MAIC |
| AE rates in stable & switch |  |  |  |  |
| Weight gain-AOM | 2.107% | 1.896%~2.318% | Beta | [8] |
| Weight gain-PP1M | 3.335% | 3.002%~3.669% | Beta | [8] |
| Weight gain-Switch | 3.477% | 3.129%~3.825% | Beta | [9] |
| Prolactin-related -AOM | 0.000% | 0.000%~0.000% | Beta | [8] |
| Prolactin-related -PP1M | 1.021% | 0.919%~1.123% | Beta | [8] |
| Prolactin-related-Switch | 0.450% | 0.405%~0.495% | Beta | [9] |
| EPS-related -AOM | 1.029% | 0.926%~1.132% | Beta | [8] |
| EPS-related -PP1M | 0.917% | 0.825%~1.008% | Beta | [8] |
| EPS-related -Switch | 0.370% | 0.333%~0.407% | Beta | [9] |
| **Costs inputs** |  |  |  |  |
| Drug acquisition costs  (per cycle) |  |  |  |  |
| AOM (400mg) | 257.619 | 231.857-283.381 | Gamma | Assumption |
| PP1M (75mg) | 208.948 | 188.053-229.843 | Gamma | [10] |
| Aripiprazole tablets (10mg) | 0.173 | 0.155-0.190 | Gamma | [10] |
| Paliperidone extended-release tablets (3mg) | 2.826 | 2.543-3.109 | Gamma | [10] |
| Oral antipsychotics (market share-weighted prices of 10 drugs) | 19.655 | 17.689-21.620 | Gamma | [10] |
| Oral clozapine (25mg) | 0.007 | 0.006-0.008 | Gamma | [10] |
| Drug and disease management costs  (per cycle) |  |  |  |  |
| Acute (AOM) | 299.904 | 269.914-329.895 | Gamma | Documents of the five provincial health insurance bureaux^a^ |
| Acute (PP1M) | 300.401 | 270.361-330.441 | Gamma | Documents of the five provincial health insurance bureaux |
| Stable | 49.945 | 44.951-54.940 | Gamma | Documents of the five provincial health insurance bureaux |
| Relapse (inpatient) | 317.349 | 285.614-349.084 | Gamma | Documents of the five provincial health insurance bureaux |
| Relapse (outpatient) | 109.586 | 98.628-120.545 | Gamma | Documents of the five provincial health insurance bureaux |
| Switch | 482.951 | 434.656-531.247 | Gamma | Documents of the five provincial health insurance bureaux |
| Adverse event management costs  (once) |  |  |  |  |
| Weight gain | 0.281 | 0.252-0.309 | Gamma | [10] |
| prolactin-related | 35.937 | 32.343-39.531 | Gamma | [10] |
| EPS-related | 1.397 | 1.257-1.536 | Gamma | [10] |
| **Utility inputs** |  |  |  |  |
| Health state utility |  |  |  |  |
| Acute | 0.568 | 0.511-0.625 | Beta | Mapping |
| Stable | 0.890 | 0.650-0.919 | Beta | [11] |
| Relapse(outpatient) | 0.659 | 0.270-0.604 | Beta | [11] |
| Relapse(inpatient) | 0.490 | 0.460-0.762 | Beta | [11] |
| Switch | 0.575 | 0.517-0.632 | Beta | Assumption |
| AE-related disutility |  |  |  |  |
| Weight gain | -0.090 | -0.081--0.099 | Gamma | [12] |
| prolactin-related | -0.087 | -0.078--0.096 | Gamma | [12] |
| EPS-related | -0.256 | -0.230--0.282 | Gamma | [12] |

a: Average the costs of medical resource utilization projects published by 5 provincial governmental, sum the projects costs in each state.

**Appendix 3 Matching-Adjustment Indirect Comparison**

**3.1 Introduction to AOM** **Phase III Trial (NCT03172871)**

NCT03172871 was a randomized, double-blind, non-inferiority study that recruited 510 patients from May 2017 to April 2019 at 15 trial sites in China. Patients were randomized into groups to receive AOM 400 mg or oral aripiprazole 10-20 mg, with a total of 428 cases in the Full analysis set (FAS), of which 213 cases were in the AOM group; a total of 319 cases in the Per-protocol set (PPS), of which 158 cases were in the AOM group; and a total of 434 cases in the Safety analysis set (SS), of which 217 cases were in the AOM group.

The study timeframe included a 13-day screening period, a 12-week acute treatment period, and a 14 (± 2) day safety follow-up period. The primary endpoint was the change in PANSS total score from baseline to week 10, and secondary endpoints included the responder rate (percentage of patients with ≥30% reduction in PANSS total score from baseline to week 10). Safety assessments included adverse events that occurred during treatment, including extrapyramidal symptoms, body weight, and prolactin-related adverse events, which were of particular interest to the trial, and therefore only these three adverse events were considered in the markov model. The clinical trial only publicly reported the results of the 10th week of the efficiency, the 12th week of the results of the subject group according to the individual level data provided by the sponsor (Otsuka Pharmaceutical Co., Ltd.) statistics, the results of the trial are shown in the Supplementary Table 5.

Supplementary Table 5 AOM Phase III Trial Results

|  | **Endpoint** | | **AOM** |
| --- | --- | --- | --- |
| Efficacy indicators  （PPS set，N=158） | Responder rate | | 88.6% |
| Safety indicators  （SS set，N=217） | Adverse event rate | EPS-related | 39.6% |
|  |  | Weight gain | 18.4% |
|  |  | Prolactin-related | 5.1% |

**3.2 Introduction to PP1M Phase Ⅳ Trial**

The PP1M China single-arm trial was an open-label, single-arm study that recruited 652 patients from October 2012 to November 2013 at 22 trial sites in China. Patients received intramuscular injections of PP1M 150 mg on day 1, PP1M 100 mg on day 8, followed by monthly injections of PP1M 75 mg at a maintenance dose. There were 610 patients in the FAS set, 444 patients in the PPS set, and 616 patients in the SS set.

The study timeframe included a 1-week screening period, a 13-week acute treatment period, and a 1-year follow-up period. The primary endpoint was responder rate (percentage of patients with ≥30% reduction in PANSS total score from baseline to week 13). Secondary endpoints included PANSS total score from baseline to the end of week 13, and PANSS subscale scores. Safety assessments included extrapyramidal symptoms, body weight, and prolactin-related adverse events. The results of the study are shown in the Supplementary Table 6.

Supplementary Table 6 PP1M Phase Ⅳ Trial Results

|  | **Endpoint** | | **PP1M（13 week）** | **PP1M（12 week）** |
| --- | --- | --- | --- | --- |
| Efficacy indicators  （PPS set，N=444） | Responder rate | | 84.50% | 82.11% |
| Safety indicators  （SS set，N=616） | Adverse event rate | EPS-related | 11.90% | 11.04% |
|  |  | Weight gain | 7.80% | 7.22% |
|  |  | Prolactin-related | 1.50% | 1.39% |

Time frame for events reported in original literature: 13 weeks, converted to 12-week event rate in acute phase

rate$=-\frac{\ln\left( 1-p1 \right)}{t1}$，p2$=1-e^{-rt2}$

p1: probability of an event occurring within the time frame of the study as reported in the original literature, t1: Time frame for events reported in the original literature, p2: transition probability, t2: cycle period

**3.3 Baseline matching**

According to the NICE guidelines,[13] the unanchored MAIC should be included in all variables for matching, so in this study, the MAIC was included in all baseline characteristics for matching, so that the baseline of the population in the AOM and PP1M groups was consistent. The baseline characteristics of the population before and after matching are shown in the Supplementary Table 7.

Supplementary Table 7 **MAIC baseline**

| Baseline | **AOM**  **(pre-adjusted)** | **AOM**  **(adjusted)** | **PP1M** |
| --- | --- | --- | --- |
| Age (years) | 34.6 | 31.5 | 31.5 |
| Male(%) | 31.6% | 55% | 55% |
| BMI (kg/m^2^) | 23.9 | 23.2 | 23.2 |
| Weight（kg） | 63.7 | 64.4 | 64.4 |
| Ethnicity-Han（%） | 96.2% | 99% | 99% |
| PANSS total score | 89.2 | 91.8 | 91.8 |
| CGI-S total score | 5.3 | 5.3 | 5.3 |
| PSP total score | 45.9 | 44.9 | 44.9 |

Supplementary Table 8 MAIC results

|  | **Endpoint** | | **AOM**  **(pre-adjusted)** | **AOM**  **(adjusted)** | **PP1M** |
| --- | --- | --- | --- | --- | --- |
| Efficacy indicators  （PPS set） | Responder rate | | 88.6%  （N=158） | 90.5%  （N=126） | 82.11%  （N=444） |
| Safety indicators  （SS set） | Adverse event rate | EPS-related | 39.6%  （N=217） | 40.6%  （N=155） | 11.04%  （N=616） |
|  |  | Weight gain | 18.4%  （N=217） | 23.2%  （N=155） | 7.22%  （N=616） |
|  |  | Prolactin-related | 5.1%  （N=217） | 1.3%  （N=155） | 1.39%  （N=616） |

**Appendix 4 Meta-analysis of relapse rate**

**4.1 Literature search strategies**

Last update: April 1^st^,2024

We searched the electronic databases PubMed, MEDLINE, EMBAS, ClinicalTrials, The Cochrane Library, Web of Science, CNKI, collected AOM and PP1M related randomized controlled trials between database inception and April 2024.We included the following words：aripiprazole, paliperidone, long-acting injectable (LAI), schizophrenia, randomized controlled trial. Medical Subject Headings (MeSH) combined with free words were used to construct the search formula:

**Web of science:**

(aripiprazole OR paliperidone) (Topic) and (long-acting OR long-acting injectable OR intramuscular) (Topic) and (schizophrenia OR Schizophrenic Disorders) (Topic) and (Randomized controlled trial OR controlled clinical trial)

**Cochrane:**

aripiprazole OR paliperidone in Title Abstract Keyword AND long-acting OR long-acting injectable OR intramuscular in Title Abstract Keyword AND schizophrenia OR Schizophrenic Disorders in Title Abstract Keyword AND Randomized controlled trial OR controlled clinical trial in Title Abstract Keyword

**PUBMED：**

#1 "aripiprazole"[Title/Abstract] OR "paliperidone"[Title/Abstract]

#2 "long-acting"[Title/Abstract] OR "long acting injectable"[Title/Abstract] OR "intramuscular"[Title/Abstract]

#3 "schizophrenia"[Title/Abstract] OR "schizophrenic disorders"[Title/Abstract]

#4"randomized controlled trial"[Title/Abstract] OR "controlled clinical trial"[Title/Abstract]

#5 #1 AND #2 AND #3 AND #4

In addition, to ensure the comprehensiveness of the retrieved literature, reviews and meta-analyses related to anti-schizophrenia containing AOM and PP1M were searched to supplement the inclusion of relevant references cited by them.

Inclusion and exclusion criteria were developed in accordance with the PICOS(population, intervention, comparators, outcomes, study design) principle, and the retrieved literature was screened with the following Supplementary Table 7 of nerfing criteria and the nerfing process as shown in the Supplementary Figure 1.

Supplementary Table 9 Inclusion and exclusion criteria of meta analysis

| **Inclusion** | **Exclusion** |
| --- | --- |
| Population: patients with schizophrenia diagnosed after systematic diagnosis. | Duplicate document |
| Intervention: aripiprazole once-monthly or  paliperidone palmitate once-monthly | Non-Chinese and English literature |
| Comparators：limitless | Reviews, meta-analyses, manuscript, letters, conference reports, editorials, observational, retrospective, mirror, real-world studies |
| Outcomes：relapse rate |  |
| Study design：only randomized controlled trials | Non-study diseases: mental illnesses other than schizophrenia |
| - | Non-study drug: intervention or comparators drug does not contain aripiprazole once-monthly or  paliperidone palmitate once-monthly but other specifications |
| - | Non-therapeutic safety studies: pharmacokinetic studies, biochemical pathology studies, etc. |


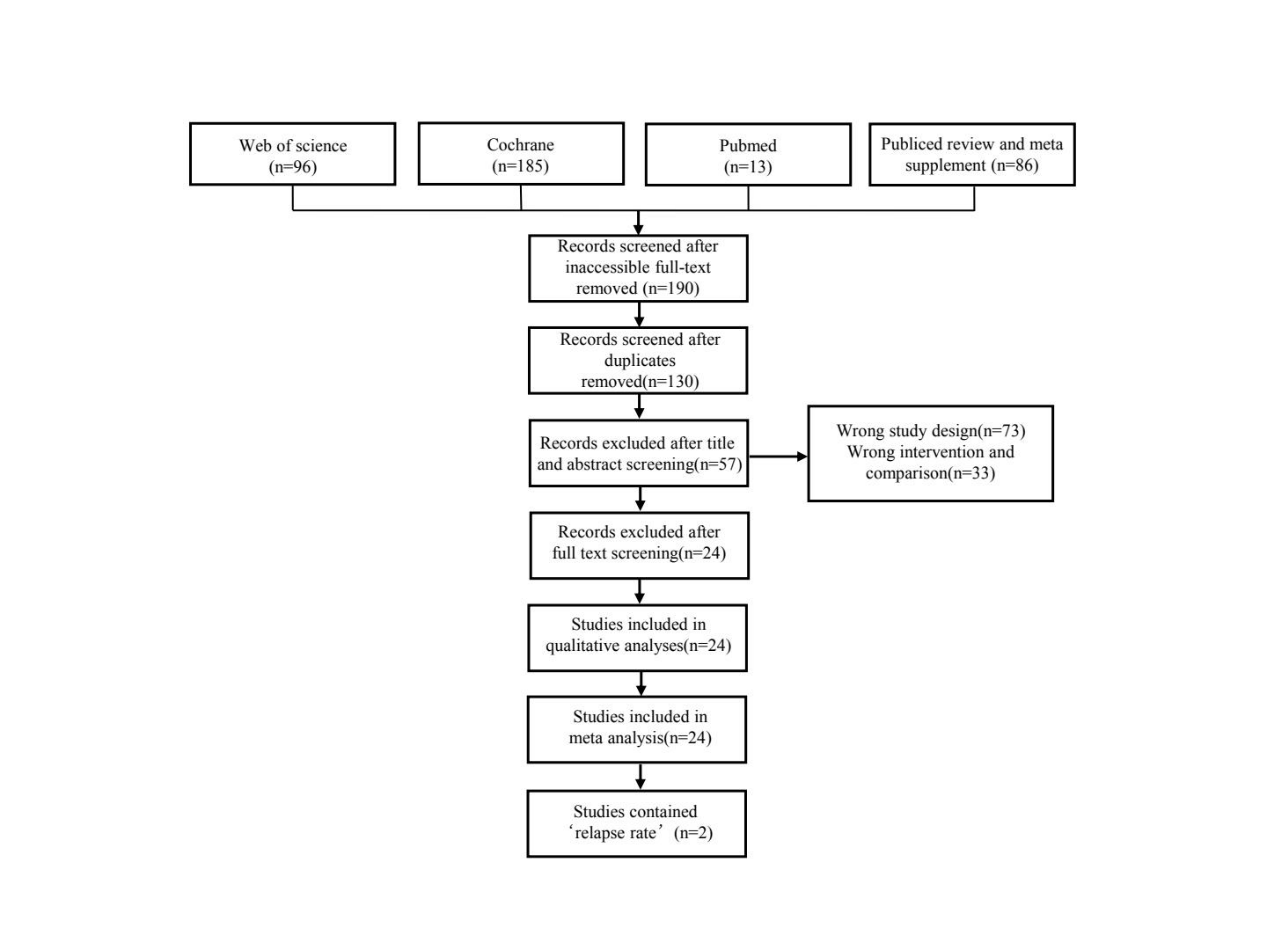


Supplementary Figure 1 Meta flow chart

Supplementary Table 10 List of included studies

| **Study author** | **Time horizon** | **Population** | **Intervention** | **Comparison** | **Primary endpoint** |
| --- | --- | --- | --- | --- | --- |
| John M Kane  2012[14] | 52 weeks | Patients with schizophrenia diagnosed according to DSM-IV-TR | AOM（N=269） | Placebo | Relapse rate |
| David Hough 2010[15] | 86 weeks | Patients with schizophrenia diagnosed according to DSM-IV-TR | PP1M（N=206） | Placebo | Relapse rate |

**4.2 Meta results**

A total of 380 papers were searched, and after screening, only 2 papers reported relapse rate data, one AOM versus placebo study and one PP1M versus placebo study. Therefore, an indirect comparison of the relapse rates of AOM and PP1M was performed using placebo as an anchor, and the results are shown in the Supplementary Table 9 below. The relapse rates for the 3-month time frame of the original literature study were converted to 1-month event rates for the modelling period.

Supplementary Table 11 Meta results

|  | **Relapse rate（3 months）** | **Relapse rate（1 months）** |
| --- | --- | --- |
| AOM | 10% | 3.45% |
| PP1M | 14.47% | 4.99% |
| RR（95%CI） | 0.691(0.407,1.173) | |

**Appendix 5 Clinical research**

In order to better understand the utilization of healthcare resources and disease management of patients with schizophrenia in clinical practice in China, this study conducted two expert surveys, online and offline, respectively. The online research took the form of questionnaires and the offline research took the form of interviews, as follows:

**5.1Online Survey Research**

(1) Survey Content

A structured physician survey questionnaire was designed to obtain relevant cost information, verify the model structure and assumptions, and confirm the accuracy of related information. The survey questionnaire mainly covers the following contents:

①Special Examination Items in Psychiatry

This includes the specific types and frequencies of psychiatric scales required for patients during their first visit, relapse, and stable phase, as well as the duration of psychiatric care and nursing for mentally ill patients, and the duration of antipsychotic drug monitoring.

②Routine Examination Items

This includes the examination items and their frequencies that need to be conducted in daily patient management, such as complete blood count, urine routine, electrocardiogram, etc.

③Adverse Event Management

This involves the adverse events that may occur with the use of AOM or PP1M by patients, as well as the corresponding management methods and duration of treatment.

(2) Survey Participants

In this study, physicians were selected based on their level and diagnostic experience. A total of six clinical experts from tertiary hospitals in Shanghai, Jiangsu, Beijing, Zhejiang, and Liaoning were surveyed. All participating physicians were associate chief physicians or above with many years of diagnostic and treatment experience, ensuring good representativeness.

(3) Processing of results

For the processing of the questionnaire results, numerical responses (e.g., length of time between examinations, number of examinations, etc.) were taken as the average of each expert, and textual responses (e.g., treatment of adverse events) were taken as the general consensus of each expert.

**5.2Offline Interview Research**

(1) Interview Content

To understand the circumstances under which patients with schizophrenia may switch medications to the next line of treatment, a 1-hour interview was conducted with each specialist. The main topics covered include the reasons for schizophrenia patients to move to the next line of treatment after taking AOM or PP1M, the proportion of patients receiving the next line of treatment, and the commonly used drugs for this treatment.

(2) Research Participants

Five clinical experts from the Guangzhou Mental Health Center, Center for Disease Control and Prevention, hospitals, and one expert from the Nanjing Brain Hospital were interviewed.

**Appendix 6 Cost inputs**

If the drugs are in the medical insurance catalogue, the medical insurance payment standard will be adopted, if the drugs are in the centralized procurement catalogue by the state organization, the highest centralized procurement price will be adopted, and the prices of other drugs will be obtained from the Menet.com. Drug dosage refer to the instruction.

**6.1 Costs of drug**

(1) Costs of AOM and PP1M for the treatment period

The current price of AOM in China is US$418.140 /400mg, considering the price reduction for access to health insurance, an assumption price of US$257.619/400mg was set in this study.

Supplementary Table 12 Costs of AOM

| **Generic name** | **Brand name** | **Manufacturer** | **Specification** | **Dosage** | **Price（**US$**）** | **Costs per cycle（**US$**）** | **Reference** |
| --- | --- | --- | --- | --- | --- | --- | --- |
| Aripiprazole for Injection | Abilify | Otsuka | 400mg | 400mg/  month | 257.619 | 257.619 | Assumption |

Supplementary Table 13 Costs of PP1M

| **Generic name** | **Brand name** | **Manufacturer** | **Specification** | **Dosage** | **Price（**US$**）** | **Initial**  **treatment（**US$**）** | **Maintenance treatment（**US$**）** | **Reference** |
| --- | --- | --- | --- | --- | --- | --- | --- | --- |
| Paliperidone Palmitate Injection |  | Qilu Pharmaceutical | 0.75ml:75mg | Day1: 150mg,  Day8:  100mg, Maintenance: 75mg/  month | 122.147 | 359.8950 | 122.147 | Menet.com |
|  |  |  | 1ml:100mg |  | 152.242 |  |  |  |
|  |  |  | 1.5ml:150mg |  | 207.653 |  |  |  |
|  | Invega Sustenna | Johnson & Johnson | 0.75ml:75mg |  | 208.948 | 615.637 | 208.948 |  |
|  |  |  | 1ml:100mg |  | 260.425 |  |  |  |
|  |  |  | 1.5ml:150mg |  | 355.211 |  |  |  |
|  |  | Original generic weighted price^a^ | 0.75ml:75mg |  | 208.948 | 615.480 | 208.948 |  |
|  |  |  | 1ml:100mg |  | 260.341 |  |  |  |
|  |  |  | 1.5ml:150mg |  | 355.139 |  |  |  |

a:Market share，Johnson & Johnson：99.53%，Qilu：0.47%(Reference from Menet.com)

b: Menet.com: https://www.menet.com.cn/

(2) Costs of oral AOM tablets and PP1M extended-release tablets for the tolerance period

Supplementary Table 14 Costs of oral AOM tablets and PP1M extended-release tablets

| **Generic name** | **Specification** | **Dosage** | **Price (**US$**)** | **Costs per cycle (**US$**)** | **Reference** |
| --- | --- | --- | --- | --- | --- |
| Oral AOM tablets | 10mg | 10mg/day | 0.173 | 2.934（17days） | Centralized purchasing price^a^ |
| PP1M extended-release tablets | 3mg | 3mg/day | 2.826 | 8.478（3days） | Menet.com^b^ |

a: Centralized purchasing price: Shanghai Sunshine Pharmaceutical Purchasing Network: https://www.smpaa.cn/

b: Menet.com: https://www.menet.com.cn/

(3) Cost of medicines in the acute state to the switch state

Supplementary Table 15 Cost of medicines in the top 10 market shares on Menet

| **Generic name** | **Specification** | **Price (**US$**)** | **Costs per year(**US$**)** | **Market share** | **Normalization** | **Reference** |
| --- | --- | --- | --- | --- | --- | --- |
| olanzapine tablets | 10mg | 1.353 | 493.714 | 18.38% | 12.32% | Centralized purchasing price |
| paliperidone extended-release tablets | 6mg | 4.998 | 1824.283 | 14.29% | 2.59% | Menet.com |
| quetiapine fumarate tablets | 100mg | 0.159 | 287.813 | 13.39% | 15.40% | Centralized purchasing price |
| aripiprazole tablets | 10mg | 0.173 | 62.995 | 6.37% | 33.46% | Centralized purchasing price |
| olanzapine orally disintegrating tablets | 5mg | 0.177 | 129.062 | 4.35% | 11.15% | Centralized purchasing price |
| quetiapine fumarate extended-release tablets | 50mg | 0.170 | 614.356 | 3.99% | 2.15% | Centralized purchasing price |
| risperidone tablets | 1mg | 0.024 | 51.834 | 2.74% | 17.49% | Centralized purchasing price |
| piperolimus hydrochloride tablets | 4mg | 0.342 | 374.895 | 2.78% | 2.45% | Menet.com |
| buprenorphine tablets | 4mg | 0.655 | 478.349 | 2.46% | 1.70% | Menet.com |
| risperidone oral solution | 60mg | 17.960 | 653.758 | 2.52% | 1.28% | Menet.com |
| Market share weighted cost of 10 drugs(US$ per cycle) | 19.655 | | | | | (Annual cost of 10 drugs$\times$ normalised market share)$\div$12 |

(4) Cost of medicines from relapse state to switch state

Supplementary Table 16 Costs of clozapine

| **Generic name** | **Specification** | **Dosage** | **Price (**US$**)** | **Costs per cycle (**US$**)** | **Reference** |
| --- | --- | --- | --- | --- | --- |
| Clozapine tablets | 25mg | Maintenance:  0.1~0.2g (4~8 tablets) /day | 0.007 | 0.854 | Centralized purchasing price |

**6.2** **Drug and disease management costs**

The frequency of examination for each health state was obtained from expert opinion, and the unit cost of medical resources was obtained from the average price of the public prices of the 5 provinces in Guangdong[16](ranking 1%), Fujian[17](ranking 25%), Liaoning[18](ranking 50%), Tianjin[19](ranking 75%), and Qinghai[20](ranking 100%) province, ranking of China's 31 provinces according to their GDP in 2023. PP1M had one more injection than AOM in the first month (Day1: 150mg, Day8: 100mg) in the acute state, so PP1M had one more intramuscular injection cost than AOM (US$0.497, average price of five provinces), and the rest of the states had the same frequency of medical resource utilization for both.

Supplementary Table 17 Costs of medical resource utilization

| **Medical resource**  **projects** | **Inspection frequency per cycle** | | | | | | **Once cost**  **(**US$**)** |
| --- | --- | --- | --- | --- | --- | --- | --- |
|  | **Acute (AOM)** | **Acute (PP1M)** | **Stable** | **Relapse**  **(inpatient)** | **Relapse**  **(outpatient)** | **Switch** |  |
| Psychiatric clinical appraisal(times) | 1 | 1 |  |  |  |  | 17.445 |
| Hospitalization(days) | 14 | 14 |  | 14 |  | 28 | 9.317 |
| Psychiatric care(days) | 14 | 14 |  | 14 |  | 28 | 3.017 |
| Psychiatric guardianship(days) | 14 | 14 |  | 14 |  | 28 | 3.177 |
| Outpatient visits (times) |  |  | 1 |  | 2 |  | 2.273 |
| Intramuscular injection(times) | 1 | 2 | 1 | 1 | 1 |  | 0.497 |
| Antipsychotic medication monitoring (days) | 30.4375 | 30.4375 | 30.4375 | 30.4375 | 30.4375 | 30.4375 | 1.116 |
| Haematology(times) | 1 | 1 | 1 | 1 | 2 | 1 | 2.315 |
| Urine routine (times) | 1 | 1 | 1 | 1 | 2 | 1 | 0.677 |
| Hormone levels (sex hormones, prolactin, etc.) (times) | 1 | 1 |  | 1 | 2 | 1 | 1.845 |
| Electroencephalogram (times) | 1 | 1 |  | 1 | 1 | 1 | 6.816 |
| Electrocardiogram (times) | 1 | 1 |  | 1 | 1 | 1 | 3.052 |
| Total costs(US$) | 299.904 | 300.401 | 49.945 | 317.349 | 109.586 | 482.951 |  |

Supplementary Table 18 Cost of Mental Scale Examination

| **Health state** | **Mental scale projects** | **Mean interval（month）** | **Frequency of inspections per cycle (times)** | **Once cost**  **(**US$**)** | **Total cost(US$)** |
| --- | --- | --- | --- | --- | --- |
| Acute | Positive and Negative Assessment of Psychiatric Symptoms Scale (PANSS) |  | 1.00 | 6.560 | 16.157 |
|  | Clinical General Impression Scale (CGI) |  | 1.00 | 2.956 |  |
|  | Hamilton Anxiety Inventory |  | 1.00 | 3.321 |  |
|  | Hamilton Depression Scale |  | 1.00 | 3.321 |  |
| Stable | Positive and Negative Assessment of Psychiatric Symptoms Scale | 3.25 | 0.31 | 6.560 | 10.230 |
|  | Clinical General Impression Scale | 3.75 | 0.27 | 2.956 |  |
|  | Brief Psychiatric Rating Scale (BPRS) | 3.50 | 0.29 | 4.034 |  |
|  | Hamilton Anxiety Inventory | 3.00 | 0.33 | 3.321 |  |
|  | Hamilton Depression Scale | 3.33 | 0.30 | 3.321 |  |
|  | Drug Side Effect Scale | 2.74 | 0.36 | 2.743 |  |
|  | Tardive Dyskinesia Rating Scale | 4.50 | 0.22 | 3.157 |  |
|  | Rating Scale for Extrapyramidal Side Effects | 2.65 | 0.38 | 2.806 |  |
|  | Abnormal Involuntary Movement Scale | 2.00 | 0.50 | 2.806 |  |
| Relapse | Positive and Negative Assessment of Psychiatric Symptoms Scale | 0.58 | 1.71 | 6.560 | 51.048 |
|  | Clinical General Impression Scale | 0.54 | 1.86 | 2.956 |  |
|  | Brief Psychiatric Rating Scale (BPRS) | 0.49 | 2.05 | 4.034 |  |
|  | Hamilton Anxiety Inventory | 0.67 | 1.49 | 3.321 |  |
|  | Hamilton Depression Scale | 0.86 | 1.16 | 3.321 |  |
|  | Drug Side Effect Scale | 0.73 | 1.37 | 2.743 |  |
|  | Tardive Dyskinesia Rating Scale | 1.11 | 0.90 | 3.157 |  |
|  | Rating Scale for Extrapyramidal Side Effects | 0.61 | 1.63 | 2.806 |  |
|  | Abnormal Involuntary Movement Scale | 0.46 | 2.17 | 2.806 |  |

**6.3** **Costs of adverse events management**

Supplementary Table 19 Costs of adverse events management

| **Adverse events** | **Treatment** | **Specification** | **Dosage** | **Price (**US$**)** | **Daily cost**  **(**US$**)** | **Number of days to deal with adverse events (days)** | Adverse event single management  **cost ($)** | **Reference** |
| --- | --- | --- | --- | --- | --- | --- | --- | --- |
| Weight gain | Metformin Hydrochloride Tablets | 0.25g | 1g/day | 0.005 | 0.020 | 14.00 | 0.281 | Centralized purchasing price |
| Prolactin-related | Bromocriptine Mesylate Tablets | 2.5mg | 15mg/day | 0.428 | 2.567 | 14.00 | 35.937 | Menet.com |
| EPS-related | Benzhexol hydrochloride tablets | 2mg | 6mg/day | 0.033 | 0.100 | 14.00 | 1.397 | Menet.com |

**Appendix 7 Mapping in acute state utility**

Supplementary Table 20 Baseline of AOM Phase III Trial (PPS)

| **Baseline** | **AOM**  **（PPS set，N=158）** |
| --- | --- |
| PANSS | 89.22 |
| PANSS positive scale | 23.39 |
| PANSS negative scale | 24.07 |
| PANSS general psychopathology | 41.77 |
| Mean age | 34.5 |
| Female(%) | 69.35% |
| Acute state utility | 0.568 |

**Appendix 8 Cost and utility components**


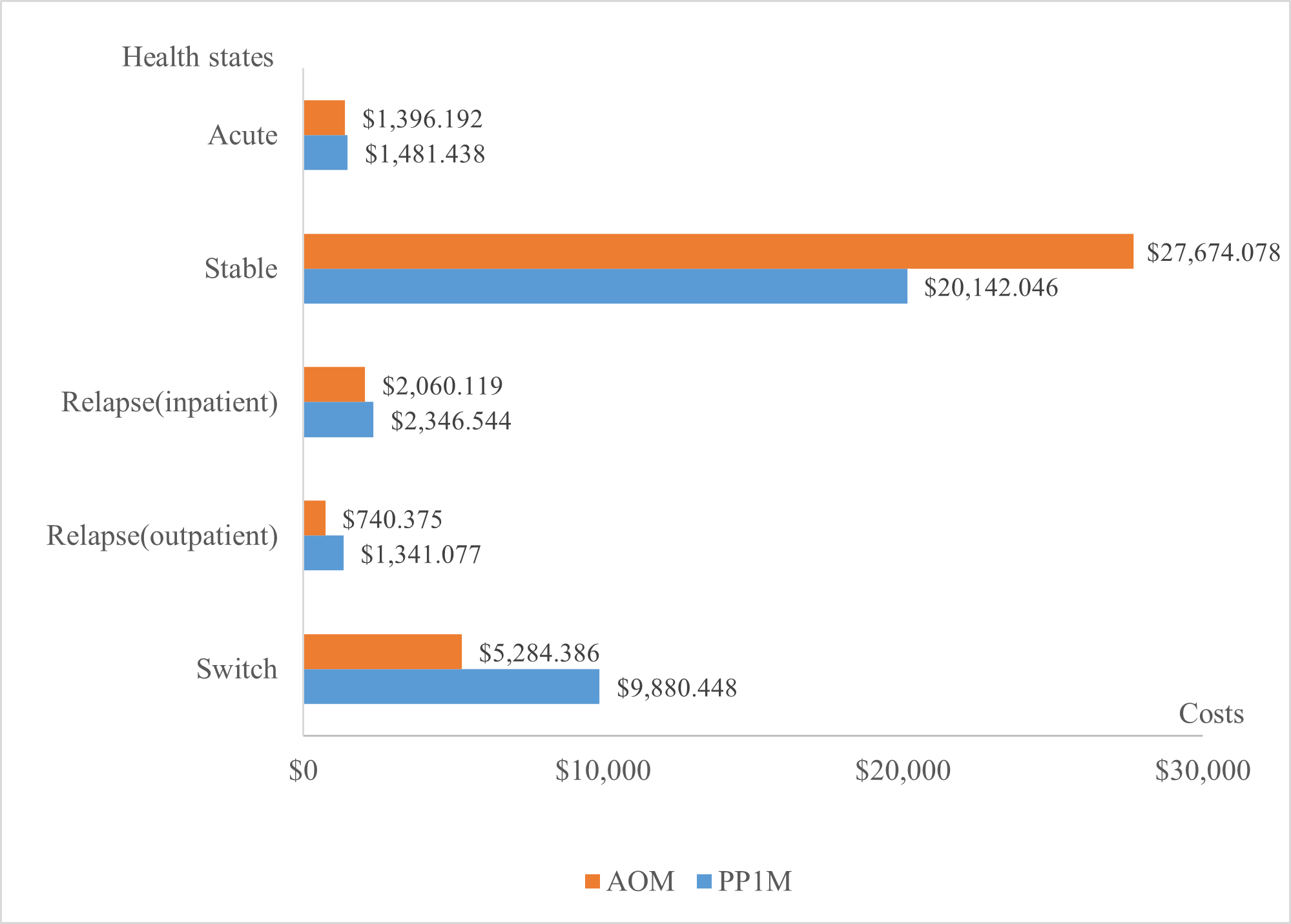


Supplementary Figure2(a) Cost components


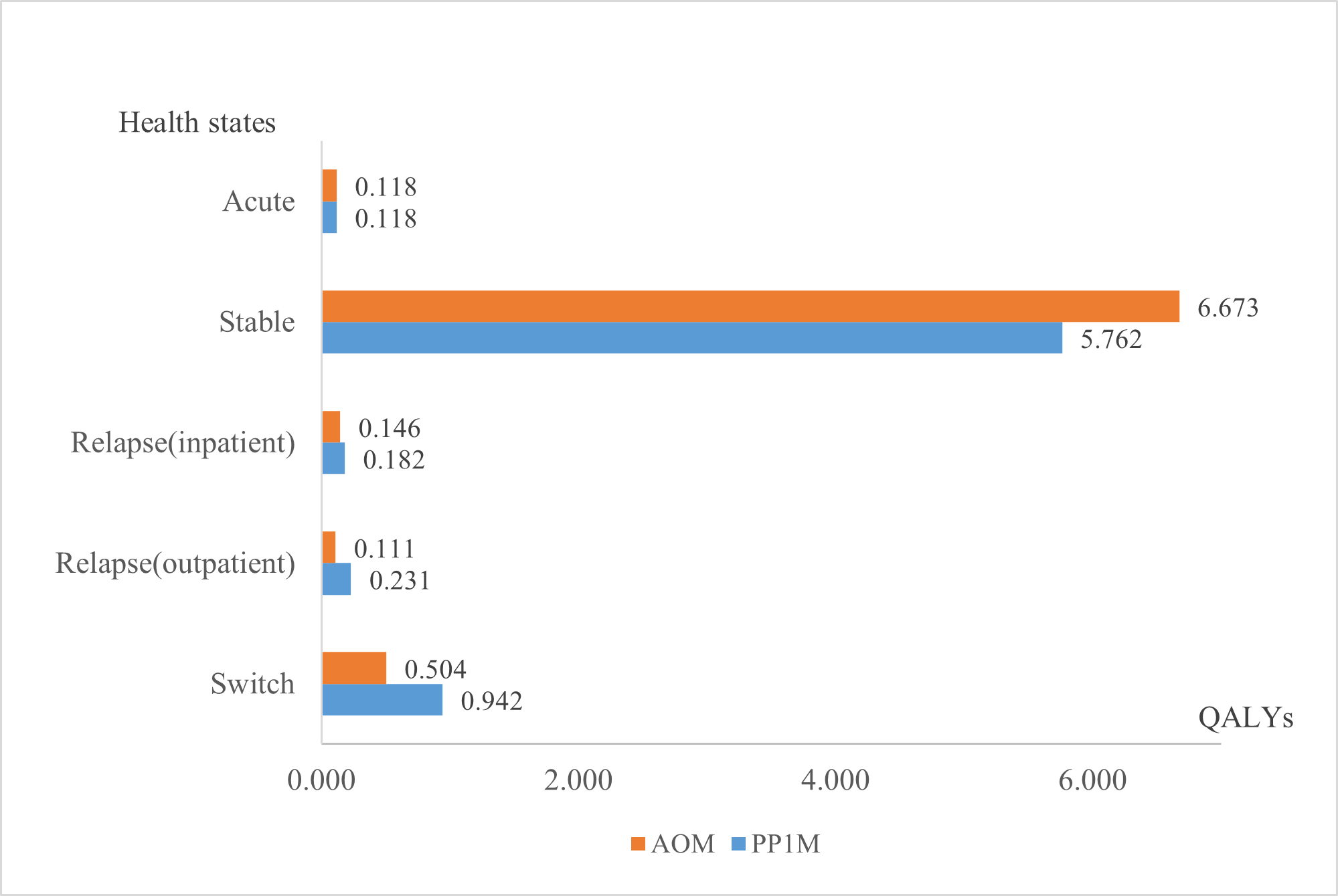


Supplementary Figure2(b) Utility components

**Appendix 9 One-way sensitivity analysis**

Tornado chart showing the top 20 parameters that impacted the results.


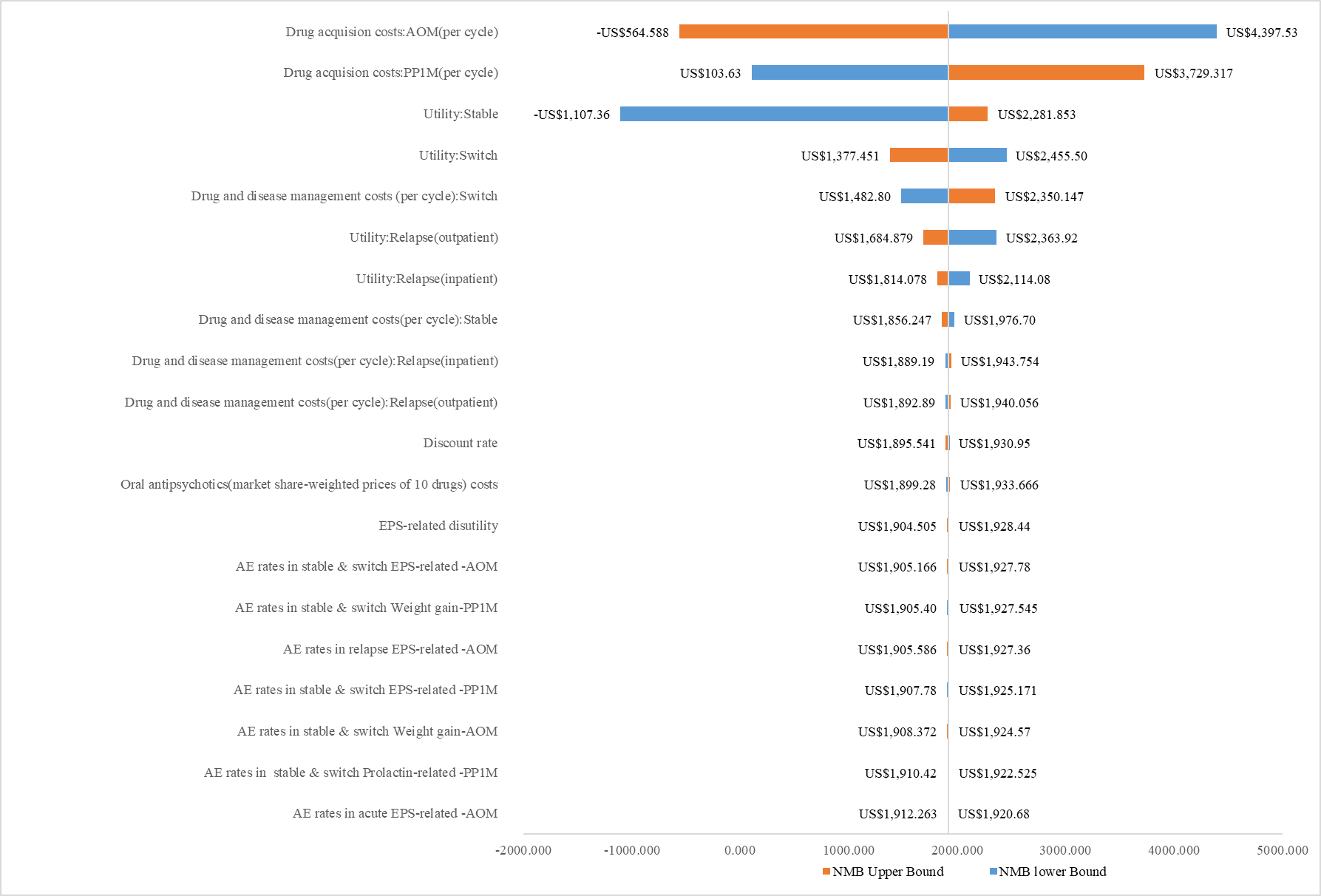


Supplementary Figure3 Tornado diagram for top20 parameters

The impact of floating the discount rate in the range of 3%-8% and other parameters in the range of ±10% on the results is ranked as follows.

Supplementary Table 21 One-way sensitivity analysis results

| **Paramaters** | **NMB lower** | **NMB upper** | **Rank** |
| --- | --- | --- | --- |
| Drug acquision costs:AOM(per cycle) | 4397.535 | -564.588 | 1 |
| Drug acquision costs:PP1M(per cycle) | 103.629 | 3729.317 | 2 |
| Utility:Stable | -1107.357 | 2281.853 | 3 |
| Utility:Switch | 2455.496 | 1377.451 | 4 |
| Drug and disease management costs (per cycle):Switch | 1482.800 | 2350.147 | 5 |
| Utility:Relapse(outpatient) | 2363.922 | 1684.879 | 6 |
| Utility:Relapse(inpatient) | 2114.077 | 1814.078 | 7 |
| Drug and disease management costs(per cycle):Stable | 1976.699 | 1856.247 | 8 |
| Drug and disease management costs(per cycle):Relapse(inpatient) | 1889.193 | 1943.754 | 9 |
| Drug and disease management costs(per cycle):Relapse(outpatient) | 1892.891 | 1940.056 | 10 |
| Discount rate | 1930.946 | 1895.541 | 11 |
| Oral antipsychotics(market share-weighted prices of 10 drugs) costs | 1899.280 | 1933.666 | 12 |
| EPS-related disutility | 1928.442 | 1904.505 | 13 |
| AE rates in stable & switch EPS-related -AOM | 1927.781 | 1905.166 | 14 |
| AE rates in stable & switch Weight gain-PP1M | 1905.401 | 1927.545 | 15 |
| AE rates in relapse EPS-related -AOM | 1927.361 | 1905.586 | 16 |
| AE rates in stable & switch EPS-related -PP1M | 1907.775 | 1925.171 | 17 |
| AE rates in stable & switch Weight gain-AOM | 1924.574 | 1908.372 | 18 |
| AE rates in stable & switch Prolactin-related -PP1M | 1910.422 | 1922.525 | 19 |
| AE rates in acute EPS-related -AOM | 1920.683 | 1912.263 | 20 |
| AE rates in relapse EPS-related -PP1M | 1912.429 | 1920.518 | 21 |
| prolactin-related disutility | 1912.982 | 1919.964 | 22 |
| AE costs prolactin-related | 1913.473 | 1919.474 | 23 |
| Weight gain disutility | 1913.839 | 1919.108 | 24 |
| AE rates in relapse Weight gain-AOM | 1918.486 | 1914.460 | 25 |
| AE rates in acute EPS-related -PP1M | 1915.329 | 1917.618 | 26 |
| AE rates in relapse Weight gain-PP1M | 1915.560 | 1917.387 | 27 |
| AE rates in acute Weight gain-AOM | 1917.315 | 1915.632 | 28 |
| Paliperidone extended-release tablets (3mg) costs | 1916.049 | 1916.897 | 29 |
| AE rates in relapse Prolactin-related -PP1M | 1916.167 | 1916.780 | 30 |
| AE rates in acute Weight gain-PP1M | 1916.211 | 1916.735 | 31 |
| AE rates in relapse Prolactin-related -AOM | 1916.659 | 1916.287 | 32 |
| Aripiprazole tablets (10mg) costs | 1916.620 | 1916.327 | 33 |
| AE costs EPS-related | 1916.609 | 1916.337 | 34 |
| AE rates in acute Prolactin-related -PP1M | 1916.383 | 1916.563 | 35 |
| AE rates in acute Prolactin-related -AOM | 1916.557 | 1916.389 | 36 |
| Oral clozapine (25mg) costs | 1916.453 | 1916.493 | 37 |
| AE costs Weight gain | 1916.456 | 1916.490 | 38 |
| Acute utility | 1916.473 | 1916.473 | 39 |
| Drug and disease management costs in acute (per cycle)（AOM） | 1916.473 | 1916.473 | 40 |
| Drug and disease management costs in acute (per cycle)（PP1M） | 1916.473 | 1916.473 | 41 |
| AE rates Weight gain-Switch | 1916.473 | 1916.473 | 42 |
| Prolactin-related -AOM | 1916.473 | 1916.473 | 43 |
| AE rates Prolactin-related-Switch | 1916.473 | 1916.473 | 44 |
| AE rates EPS-related -Switch | 1916.473 | 1916.473 | 45 |

**Appendix 10 CHEERS 2022 Checklist**

CHEERS 2022 Checklist^[21]^

| **Topic** | **No.** | **Item** | **Location where item is reported** |
| --- | --- | --- | --- |
| **Title** |  |  |  |
|  | 1 | Identify the study as an economic evaluation and specify the interventions being compared. | Title |
| **Abstract** |  |  |  |
|  | 2 | Provide a structured summary that highlights context, key methods, results, and alternative analyses. | Abstract |
| **Introduction** |  |  |  |
| **Background and objectives** | 3 | Give the context for the study, the study question, and its practical relevance for decision making in policy or practice. | Abstract and Introduction |
| **Methods** |  |  |  |
| **Health economic analysis plan** | 4 | Indicate whether a health economic analysis plan was developed and where available. | Methods Study design |
| **Study population** | 5 | Describe characteristics of the study population (such as age range, demographics, socioeconomic, or clinical characteristics). | Methods Patient population |
| **Setting and location** | 6 | Provide relevant contextual information that may influence findings. | Introduction |
| **Comparators** | 7 | Describe the interventions or strategies being compared and why chosen. | Introduction |
| **Perspective** | 8 | State the perspective(s) adopted by the study and why chosen. | Introduction |
| **Time horizon** | 9 | State the time horizon for the study and why appropriate. | Methods Study design |
| **Discount rate** | 10 | Report the discount rate(s) and reason chosen. | Methods Study design |
| **Selection of outcomes** | 11 | Describe what outcomes were used as the measure(s) of benefit(s) and harm(s). | Methods Study design |
| **Measurement of outcomes** | 12 | Describe how outcomes used to capture benefit(s) and harm(s) were measured. | Methods |
| **Valuation of outcomes** | 13 | Describe the population and methods used to measure and value outcomes. | Methods Clinical inputs and transition probabilities |
| **Measurement and valuation of resources and costs** | 14 | Describe how costs were valued. | Methods Costs inputs |
| **Currency, price date, and conversion** | 15 | Report the dates of the estimated resource quantities and unit costs, plus the currency and year of conversion. | Methods Study design |
| **Rationale and description of model** | 16 | If modelling is used, describe in detail and why used. Report if the model is publicly available and where it can be accessed. | Methods Model structure |
| **Analytics and assumptions** | 17 | Describe any methods for analysing or statistically transforming data, any extrapolation methods, and approaches for validating any model used. | Methods |
| **Characterising heterogeneity** | 18 | Describe any methods used for estimating how the results of the study vary for subgroups. | Method Model Validation |
| **Characterising distributional effects** | 19 | Describe how impacts are distributed across different individuals or adjustments made to reflect priority populations. | Methods  Sensitivity analysis |
| **Characterising uncertainty** | 20 | Describe methods to characterise any sources of uncertainty in the analysis. | Methods Sensitivity analyses |
| **Approach to engagement with patients and others affected by the study** | 21 | Describe any approaches to engage patients or service recipients, the general public, communities, or stakeholders (such as clinicians or payers) in the design of the study. | Supporting information |
| **Results** |  |  |  |
| **Study parameters** | 22 | Report all analytic inputs (such as values, ranges, references) including uncertainty or distributional assumptions. | Appendix2 |
| **Summary of main results** | 23 | Report the mean values for the main categories of costs and outcomes of interest and summarise them in the most appropriate overall measure. | Results and Table2 |
| **Effect of uncertainty** | 24 | Describe how uncertainty about analytic judgments, inputs, or projections affect findings. Report the effect of choice of discount rate and time horizon, if applicable. | Sensitivity analysis, Scenario analyses |
| **Effect of engagement with patients and others affected by the study** | 25 | Report on any difference patient/service recipient, general public, community, or stakeholder involvement made to the approach or findings of the study | Not reported |
| **Discussion** |  |  |  |
| **Study findings, limitations, generalisability, and current knowledge** | 26 | Report key findings, limitations, ethical or equity considerations not captured, and how these could affect patients, policy, or practice. | Discussion |
| **Other relevant information** |  |  |  |
| **Source of funding** | 27 | Describe how the study was funded and any role of the funder in the identification, design, conduct, and reporting of the analysis | End of manuscript |
| **Conflicts of interest** | 28 | Report authors conflicts of interest according to journal or International Committee of Medical Journal Editors requirements. | End of manuscript |

**References**

1. Xiao L, Zhao Q, Li AN, Sun J, Wu B, Wang L, et al. Efficacy and safety of aripiprazole once-monthly versus oral aripiprazole in Chinese patients with acute schizophrenia: a multicenter, randomized, double-blind, non-inferiority study. Psychopharmacology (Berl). 2022;239(1):243-51.

2. Ishigooka J, Nakamura J, Fujii Y, Iwata N, Kishimoto T, Iyo M, et al. Efficacy and safety of aripiprazole once-monthly in Asian patients with schizophrenia: a multicenter, randomized, double-blind, non-inferiority study versus oral aripiprazole. Schizophrenia research. 2015;161(2-3):421-8.

3. Edlin R, McCabe C, Hulme C, Hall P, Wright J. Cost Effectiveness Modelling for Health Technology Assessment A Practical Course. Springer International2015.

4. Druais S, Doutriaux A, Cognet M, Godet A, Lançon C, Levy P, et al. Cost Effectiveness of Paliperidone Long-Acting Injectable Versus Other Antipsychotics for the Maintenance Treatment of Schizophrenia in France. Pharmacoeconomics. 2016;34(4):363-91.

5. Mason K, Barnett J, Pappa S. Effectiveness of 2-year treatment with aripiprazole long-acting injectable and comparison with paliperidone palmitate. Therapeutic Advances in Psychopharmacology. 2021;11:20451253211029490.

6. Statistics NBo. CHINA STATISTICAL YEARBOOK 2023 [cited 2024 09]. Available from: <https://www.stats.gov.cn/sj/ndsj/2023/indexch.htm>.

7. Correll CU, Solmi M, Croatto G, Schneider LK, Rohani-Montez SC, Fairley L, et al. Mortality in people with schizophrenia: a systematic review and meta-analysis of relative risk and aggravating or attenuating factors. World Psychiatry. 2022;21(2):248-71.

8. Naber D, Hansen K, Forray C, Baker RA, Sapin C, Beillat M, et al. Qualify: a randomized head-to-head study of aripiprazole once-monthly and paliperidone palmitate in the treatment of schizophrenia. Schizophr Res. 2015;168(1-2):498-504.

9. Huhn M, Nikolakopoulou A, Schneider-Thoma J, Krause M, Samara M, Peter N, et al. Comparative efficacy and tolerability of 32 oral antipsychotics for the acute treatment of adults with multi-episode schizophrenia: a systematic review and network meta-analysis. Lancet. 2019;394(10202):939-51.

10. MENET [Internet]. Available from: <https://db.menet.com.cn/>.

11. Zhou J, Millier A, François C, Aballéa S, Toumi M. Systematic review of utility values used in the pharmacoeconomic evaluations for schizophrenia: implications on cost-effectiveness results. J Mark Access Health Policy. 2019;7(1):1648973.

12. Briggs A, Wild D, Lees M, Reaney M, Dursun S, Parry D, et al. Impact of schizophrenia and schizophrenia treatment-related adverse events on quality of life: direct utility elicitation. Health Qual Life Outcomes. 2008;6:105.

13. Phillippo DM AA, Dias S, et al. . NICE DSU technical support document 18: methods for population-adjusted indirect comparisons in submission to NICE. 2016.

.

14. Kane JM, Sanchez R, Perry PP, Jin N, Johnson BR, Forbes RA, et al. Aripiprazole intramuscular depot as maintenance treatment in patients with schizophrenia: a 52-week, multicenter, randomized, double-blind, placebo-controlled study. J Clin Psychiatry. 2012;73(5):617-24.

15. Hough D, Gopal S, Vijapurkar U, Lim P, Morozova M, Eerdekens M. Paliperidone palmitate maintenance treatment in delaying the time-to-relapse in patients with schizophrenia: a randomized, double-blind, placebo-controlled study. Schizophr Res. 2010;116(2-3):107-17.

16. FOSHAN PROVINCIAL HEALTHCARE SECURITY BUREAU. Basic Medical Service Items and Prices in Foshan Public Hospitals <https://www.foshan.gov.cn/gzjg/fsylbzj/zwdt/tzgg/content/post_5601780.html2023> [cited 2024 April 1]. Available from: <https://www.foshan.gov.cn/gzjg/fsylbzj/zwdt/tzgg/content/post_5601780.html>.

17. FUJIAN PROVINCIAL HEALTHCARE SECURITY BUREAU. Medical Service Price Items for Medical Institutions in Fujian Province 2019 [cited 2024 April 1]. Available from: <https://ybj.fujian.gov.cn/zfxxgkzl/fdzdgknr/zcwj/201903/t20190319_4830769.htm#>.

18. LIAONING PROVINCIAL HEALTHCARE SECURITY BUREAU. Circular on the Establishment of Maximum Prices for Medical Service Items in Public Medical Institutions in Liaoning Province and Related Issues 2019 [cited 2024 April 1]. Available from: <https://ybj.ln.gov.cn/ybj/zfxxgk/fdzdgknr/lzyj/bbmgfxwj/D082B4EA21D54C1BAA897F109012DE50/index.shtml#>.

19. TIANJIN PROVINCIAL HEALTHCARE SECURITY BUREAU. Notice on Regulating and Adjusting the Prices of Some Medical Service Items and Medical Insurance Payment Standards 2024 [cited 2024 April 1]. Available from: <https://ylbz.tj.gov.cn/xxgk/zcfg/ybjwj/202404/t20240412_6598530.html>.

20. QINGHAI PROVINCIAL HEALTHCARE SECURITY BUREAU. 2024 Summary of Medical Service Prices for Public Medical Institutions in Qinghai Province 2024 [cited 2024 April 1]. Available from: <https://ybj.qinghai.gov.cn/2024-04/03/c_1130102713.htm>.

21. Husereau D, Drummond M, Augustovski F, de Bekker-Grob E, Briggs AH, Carswell C, et al. Consolidated Health Economic Evaluation Reporting Standards 2022 (CHEERS 2022) Explanation and Elaboration: A Report of the ISPOR CHEERS II Good Practices Task Force. Value in health: the journal of the International Society for Pharmacoeconomics and Outcomes Research. 2022;25(1).
